# Anti-inflammatory and antioxidant effects of nanoformulations composed of metal-organic frameworks delivering rutin and/or piperine natural agents

**DOI:** 10.1080/10717544.2021.1949073

**Published:** 2021-07-13

**Authors:** Khaled AbouAitah, Imane M. Higazy, Anna Swiderska-Sroda, Reda M. Abdelhameed, Stanislaw Gierlotka, Tarik A. Mohamed, Urszula Szałaj, Witold Lojkowski

**Affiliations:** a Laboratory of Nanostructures and Nanomedicine, Institute of High Pressure Physics, Polish Academy of Sciences, Warsaw, Poland; b Medicinal and Aromatic Plants Research Department, Pharmaceutical and Drug Industries Research Division, National Research Centre (NRC), Giza, Egypt; c Department of Pharmaceutical Technology, Pharmaceutical and Drug Industries Research Division, National Research Centre (NRC), Giza, Egypt; d Applied Organic Chemistry Department, Chemical Industries Research Division, National Research Centre (NRC), Giza, Egypt; e Chemistry of Medicinal Plants Department, Pharmaceutical and Drug Industries Research Division, National Research Centre (NRC), Giza, Egypt; f Faculty of Materials Science and Engineering, Warsaw University of Technology, Warsaw, Poland

**Keywords:** Delivery system, release kinetics, metal organic framework (MOF), rutin flavonoid and piperine alkaloid natural agents nanoformulations, *in vivo* antioxidant and anti-inflammatory

## Abstract

Plant-derived natural medicines have been extensively studied for anti-inflammatory or antioxidant properties, but challenges to their clinical use include low bioavailability, poor solubility in water, and difficult-to-control release kinetics. Nanomedicine may offer innovative solutions that can enhance the therapeutic activity and control release kinetics of these agents, opening the way to translating them into the clinic. Two agents of particular interest are rutin (Ru), a flavonoid, and piperine (Pip), an alkaloid, which exhibit a range of pharmacological activities that include antioxidant and anti-inflammatory effects. In this work, nanoformulations were developed consisting of two metal–organic frameworks (MOFs) with surface modifications, Ti-MOF and Zr-MOF, each of them loaded with Ru and/or Pip. Both MOFs and nanoformulations were characterized and evaluated *in vivo* for anti-inflammatory and antioxidant effects. Loadings of ∼17 wt.% for a single pro-drug and ∼27 wt.% for dual loading were achieved. The release patterns for Ru and or Pip followed two stages: a zero-order for the first 12-hour stage, and a second stage of stable sustained release. At pH 7.4, the release patterns best fit to zero-order and Korsmeyer–Peppas kinetic models. The nanoformulations had enhanced anti-inflammatory and antioxidant effects than any of their elements singly, and those with Ru or Pip alone showed stronger effects than those with both agents. Results of assays using a paw edema model, leukocyte migration, and plasma antioxidant capacity were in agreement. Our preliminary findings indicate that nanoformulations with these agents exert better anti-inflammatory and antioxidant effects than the agents in their free form.

## Introduction

Drug delivery systems (DDSs) are the main nanomedicine platforms in modern pharmaceutical research, application, and development used to control release of therapeutic agents, reduce side effects, increase bioavailability and solubility, enhance targeting, and improve therapeutic activity. Building an effective DDS requires a suitable drug vehicle/carrier, which generally fall into two types, those for organic materials (i.e. chitosan nanoparticles, liposomes) and those for inorganic materials (i.e. mesoporous silica nanoparticles) (Horcajada et al., 2006). A third, hybrid type, the inorganic–organic route, has emerged in recent decades and relies on metal–organic frameworks (MOFs) for drug delivery (Horcajada et al., 2006). MOF solids are somewhat new and have highly specific porosity and surface areas, offering the potential for many different uses, including medical applications (Chedid & Yassin, 2018). The first synthesis report for MOFs was published in 1989 (Hoskins & Robson, 1989; Horcajada et al., 2012). They consist of a crystalline network of metal-in-metal clusters or single metal ions associated with organic linkers that are strongly covalently bonded (Domingos et al., 2015; Pettinari et al., 2017) and also have been characterized as coordination polymers containing metal ions centrally, with organic linkers, or as porous coordination networks (Janiak & Vieth, 2010; Gangu et al., 2016; Santos et al., 2020). These frameworks have several notable features, including that their surface area is highly specific, the size of their pores is adjustable, and their porosity is tunable. In addition, they are flexible, low density, and thermally stable, with a distinct ordered structure, versatile functionality, good biocompatibility, and low toxicity. MOFs also can be developed using various metals and linkers (Shearer et al., 2016; Zhao 2016; Pettinari et al., 2017; Han et al., 2018; Rojas et al., 2018; Su et al., 2019; Liu et al., 2020).

Drug-loading capacity is an important prerequisite for the applicability of a DDS drug carrier. MOFs show efficient drug-loading capacity for various candidates, such as the anticancer drugs 5-fluorouracil (∼28 wt.%; Hu et al., 2020) and doxorubicin (DOX; ∼10 wt.%; Liang et al., 2018), caffeine (∼15–50 wt.%; Horcajada et al., 2007; Cunha et al., 2013a,b; Chevreau et al., 2016), magnolol (∼72%; Santos et al., 2020), ibuprofen (>49%; Lu et al., 2020), gentamicin antibiotic (19 wt.%; Soltani et al., 2018), and thymol essential component (∼4%; Wu et al., 2019). Several studies suggest that MOFs of various types show great promise in drug delivery of, e.g. anticancer, anti-inflammatory, and antibacterial agents (Dong et al., 2018; Li et al., 2019b; Nasrabadi et al., 2019; Abánades Lázaro et al., 2020; Lu et al., 2020). An important and unique feature of MOFs as carriers is their low toxicity and their enhancement of bioavailability and solubility of the drug. For example, Santos et al. (2020) reported that a Zr-based MOF enhances the bioavailability of magnolol (which is poorly soluble) and that the magnolol-loaded MOF exerted no toxicity at 2000 mg/kg in female Sprague–Dawley rats.

Natural agents remain a valuable source of medicines, and hundreds of promising agents likely remain to be discovered and evaluated for human diseases. The advantages of natural agents are their potentially greater safety, cost-effectiveness, and pharmacological versatility (Harvey, 2008; Atanasov et al., 2021). Common disadvantages to their use are poor bioavailability and water solubility, lack of targeting specificity, and difficulty with achieving controlled release. Nanomedicine technology offers a potential solution to these problems. Here, two plant-derived agents, piperine (an amide alkaloid also known as Pip) and rutin (a flavonoid known as Ru), are of special interest. Black pepper, a common spice, can yield about 6–9% pure Pip from its fruits (Damanhouri & Ahmad, 2014; Gorgani et al., 2017). Pip shows potential in exerting anti-inflammatory (Bang et al., 2009), neuroprotective (Yang et al., 2015), antioxidant (Selvendiran et al., 2003), and anti-tumor (Selvendiran et al., 2004; Do et al., 2013; Samykutty et al., 2013; Yaffe et al., 2015; Gunasekaran et al., 2017; Si et al., 2018; Yoo et al., 2019) effects and may enhance drug bioavailability (Shoba et al., 1998; Kasibhatta & Naidu, 2007). Ru, given technically as 3,30,40,5,7-pentahydroxyflavone-3-rhamnoglucoside, occurs abundantly in many plants, e.g. buckwheat, tea, citrus, and apple (Harborne, 1986). Its potential activities include anti-inflammatory, antioxidant, anti-bacterial, anti-cancer, neuroprotective, and cardioprotective effects (Guardia et al., 2001; Annapurna et al., 2009; Khan et al., 2009; Alonso-Castro et al., 2013; Al-Rejaie et al., 2013; Kamel et al., 2014; Ganeshpurkar & Saluja, 2017). Despite these fascinating pharmacological effects in *in vitro* and pre-clinical studies, Ru and Pip have yet to be examined in clinical investigations, primarily because of the inherent limitations of such agents (e.g. solubility, bioavailability, site-specific targeting, and bioavailability).

**Scheme 1.**
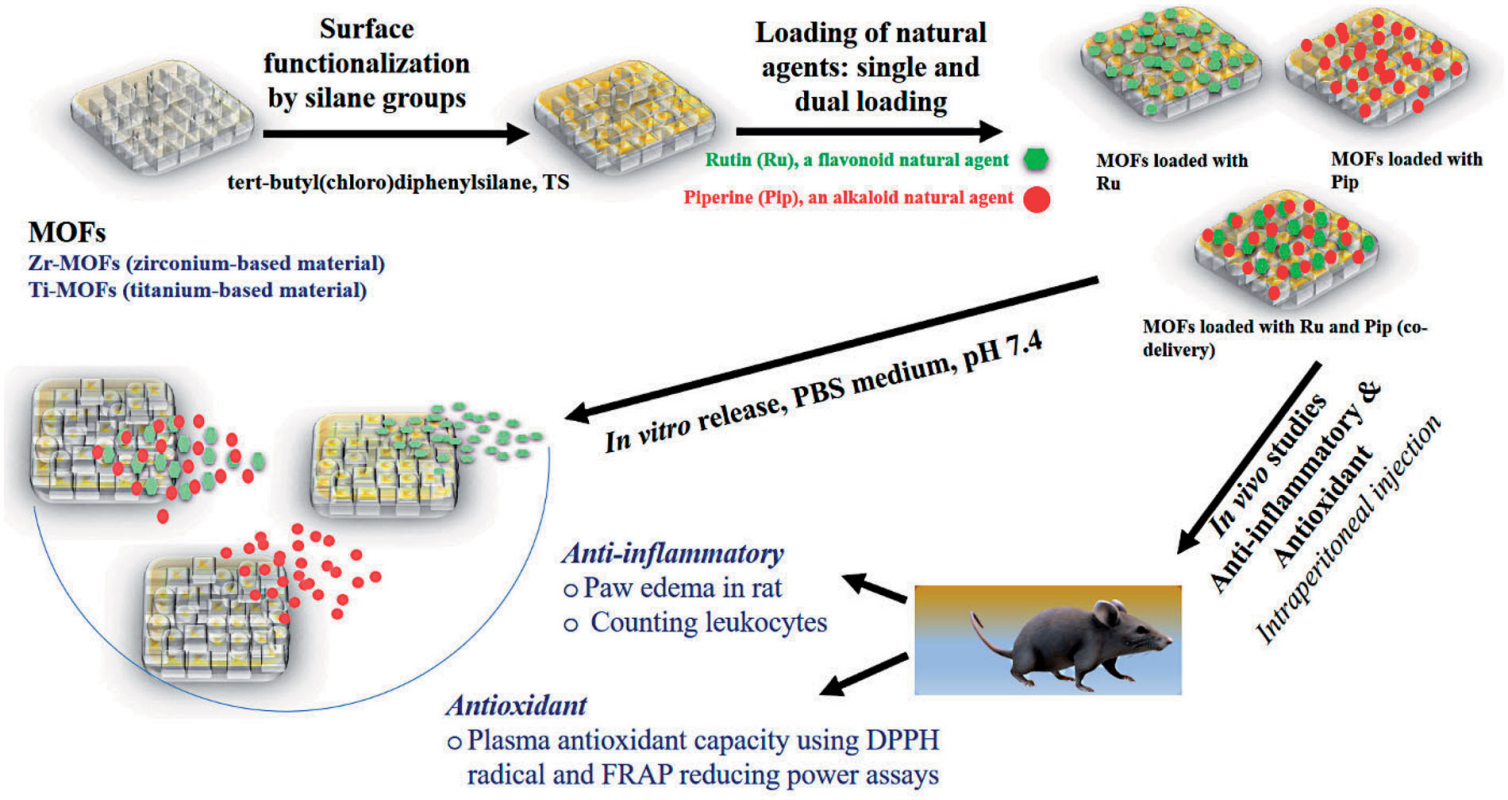
The schematic representation of the preparation of nanoformulations consisting of MOFs carrying Ru and/or Pip, and the evaluation *in vivo* for anti-inflammatory and antioxidant effects.

No delivery systems for Ru and/or Pip using MOFs have, to our knowledge, been published. Here, we describe a novel delivery system we designed that contains two types of MOFs, one zirconium-based (Zr-MOFs) and the other titanium-based (Ti-MOFs), loaded with Ru and/or Pip to yield various nanoformulations (Scheme 1). As the loading capacity of any drug or therapeutic agent is important for determining its activity and release, we also evaluated these constructs for high-loading capacity and the ability to co-deliver two drugs. In a final step, we evaluated whether the nanoformulations enhanced therapeutic efficiency compared with the effects of the free natural agents. In the *in vivo* studies, we found enhanced anti-inflammatory and antioxidant activities of the nanoformulation with each natural agent compared with either free agent alone.

## Materials and methods

### Synthesis of ZrMOF (UiO-66-COOH)

A 100 mL reaction kettle was charged with 1,2,4-benzene tricarboxylic acid (0.424 g), ZrCl_4_ (0.463 g), DMF (10 mL), demineralized water (8.8 mL), and acetic acid (12.5 mL). The reaction was carried out at 100 °C for 24 h. MOFs were separated from the reaction solution by centrifugation (10,000 rpm, 5 min × 3) and washing with methanol. Finally, the drying process was carried out under vacuum at 55 °C, and UiO-66 was obtained (Abdelhameed et al., 2018; Li et al., 2020).

### Synthesis of TiMOF (MIL-125-NH_2_)

The TiMOF was prepared according to Abdelhameed et al. (2015) as follows: 2-aminoterephthalic acid (1 g, 5.5 mmol) was dissolved in mixture of DMF/methanol (2:1, v/v). To the mixture, titanium isopropoxide (1 mL, 3.38 mmol) was added at room temperature (RT) under continuous stirring. The mixture was then kept for 24 h at 150 °C. After the solvo-thermal process, the slurry was converted to yellowish precipitate which was isolated by filtration and then washed by DMF followed by methanol. The isolated precipitate was dried under vacuum to obtain TiMOF powder.

### Surface modification of MOFs

**Figure 1.**
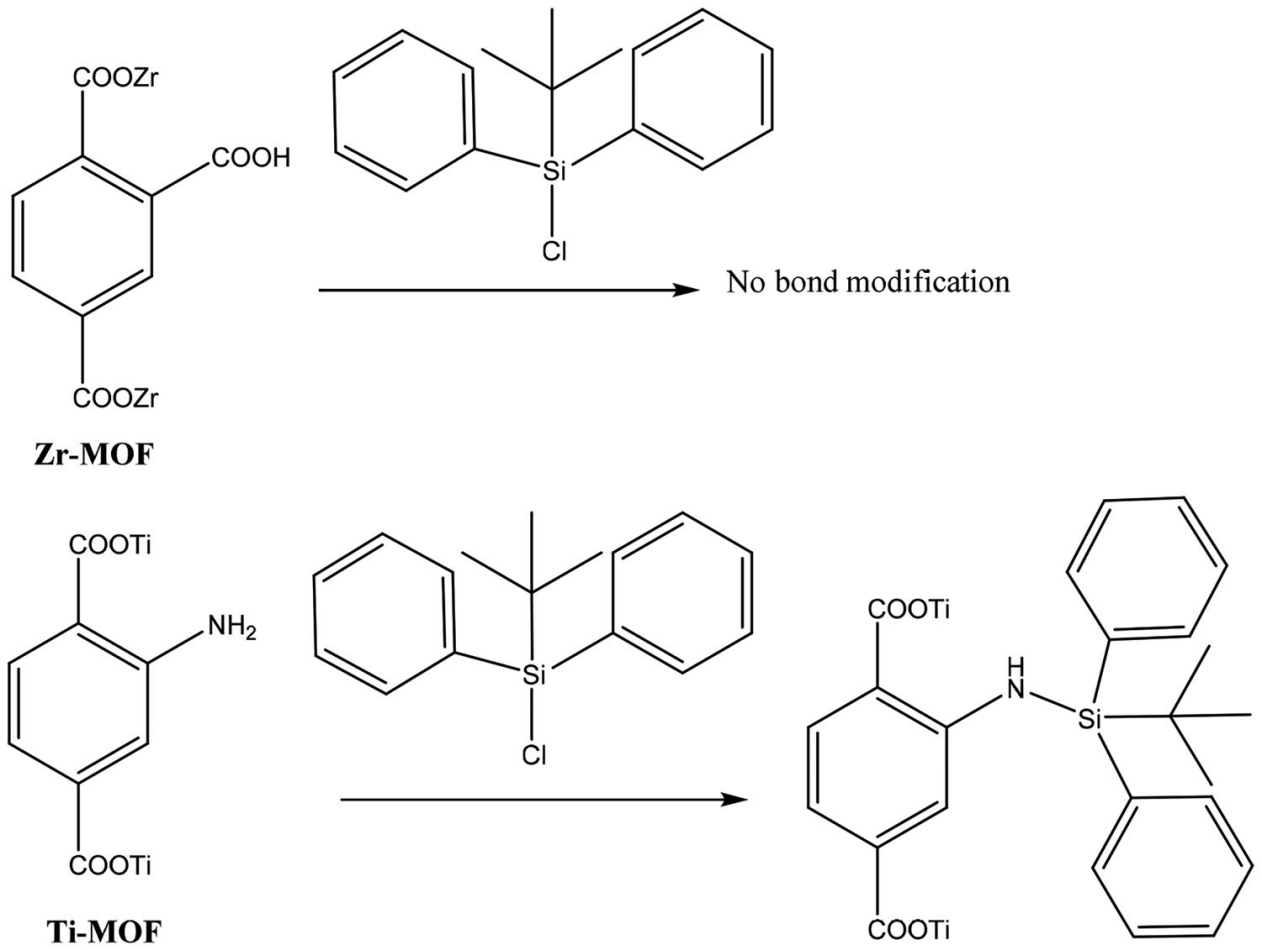
Post-synthetic modification of ZrMOFs and TiMOFs with TS silane.

ZrMOF and TiMOF materials were functionalized with silane TS groups through post-synthesis route (Figure 1). Typically, 0.5 g of MOFs was suspended in 50 mL anhydrous toluene (POCH, Gliwice, Poland) by use of sonication (water bath sonicator; Elma GmbH, Singen, Germany) for 10 minutes. Afterward, the TS silane (tert-butyl(chloro) diphenyl-silane 98%; Cross Organics, Geel, Belgium) was added to the solution drop by drop under vigorous stirring, followed by adjustment of the stirring speed to 300 rpm and maintenance of the solution at RT for 24 hours. We then washed and filtered the solution with methanol three or four times (Fisher Scientific, Loughborough, UK) and deionized water to remove un-reacted TS silane molecules with MOF particles. In a final step, the materials were dried for 24 hours at 60 °C. The resulting functionalized MOFs were designated as ZrMOFTS and TiMOFTS.

### Preparation of nanoformulations

Ru was isolated from plant material according to Zhu et al. (2017). For the isolation, 2 kg of *Punica granatum* dried powder peel was extracted in aqueous methanol (80%) three times. After evaporation of the combined extracts at 45 °C *in vacuo*, there was about ∼100 g of a dark brown residue. For the initial separation, we used hexane, CH_2_Cl_2_, EtOAc, and BuOH for liquid–liquid extraction of the crude extract. To purify the EtOAc fraction and yield Ru, we used silica gel column chromatography and Sephadex LH-20. The purity of isolated Ru was identified by NMR and HPLC techniques and the data are placed in supplementary material (Figure S1–S3). We purchased Pip from Sigma-Aldrich (St. Louis, MO).

Ru or Pip or both were loaded to the functionalized ZrMOFTS and TiMOFTS in single or dual loadings. The drug:MOF ratio was 1:2, and for dual loading, the Ru:Pip ratio was fixed at 1:1. In a typical experiment, 100 mg of Ru or Pip (single loading) or 50 mg of Ru plus 50 mg of Pip (dual loading) was dissolved in ethanol (15 mL), followed by addition to the solution of 200 mg of ZrMOFTS or TiMOFTS. After stirring (200 rpm) at RT for 24 hours, the solution was transferred to a round flask, evaporated at 60 °C in a Rotavap (Büchi, Flawil, Switzerland), and the resulting powder resuspended in deionized water, followed by another evaporation to remove unloaded Ru or Pip. This step was repeated once more to ensure removal of unloaded agent. To yield the final nanoformulations, the resulting materials were dried for 12 hours at 60 °C in an oven. The drug-loaded MOF nanoformulations were designated as ZrMOFTS-Ru, ZrMOFTS-Pip, ZrMOFTS-Ru-Pip, TiMOFTS-Ru, TiMOFTS-Pip, and TiMOFTS-Ru-Pip.

### Characterization

To observe the morphology of the materials, we used field-emission (FE) scanning electron microscopy (SEM) (Ultra Plus, Zeiss, Jena, Germany) at 3 kV with different magnifications. Before imaging, the materials were sputter coated with gold–palladium (Bal-Tech SCD 005, Balzers, Liechtenstein). To record the crystalline patterns of the materials, we used powder X-ray diffraction (XRD) (X’PertPRO System, PANalytical, Almelo, Netherlands), with CuKα radiation (40 mA and 40 kV; 2*θ* range of 5–100). For identifying the functional groups on the material surface, we used Fourier transform infrared (FTIR) spectroscopy (Bruker Optics Tensor 27, Bruker Corporation, Billerica, MA) with attenuated total reflectance (ATR, Platinium ATR-Einheit A 255, Bruker, Karlsruhe, Germany). ATR-FTIR spectra were performed at 400–4000 cm^−1^ range, with spectral resolution of 1 cm^−1^. For the simultaneous thermal analysis (STA), which was coupled with differential scanning, we used the STA 499 F1Jupiter (NETZSCH-Feinmahltechnik GmbH, Selb, Germany). Measurements were done in the temperature range RT-800 °C in a gas mixture of helium and synthetic air flowing through the furnace chamber. Before starting the experiment, the chamber was purged for 10 minutes with the same gas mixture. A similar amount of sample, approximately 10 mg, was used for all experiments. We determined the zeta potential with a NanoZS Malvern ZetaSizer (Malvern, UK) by creating a suspension of the materials in deionized water (Hydrolab, Straszyn, Poland) at 1 mg/mL, adjusted to ∼ pH 7.4 and measured at 23.5 °C. For the particle size distributions, we analyzed materials using measurements derived from dynamic light scattering (DLS) recorded at RT. Particle size and polydispersity index (PDI) for the materials were determined using the DLS technique, following reconstitution in distilled water. CNH contents were determined by LECO CHNS-932 element analyzer (Leco-Corporation, St. Joseph, MI). The metal concentrations on prepared materials were analyzed with an atomic absorption spectrophotometer (Perkin-Elmer Analyst 200 AAS, Waltham, MA).

### Entrapment efficiency (EE) and total drug content in nanoformulations

*EE*: Accurately weighed nanoformulations (5 mg) were suspended in 10 mL of ethanol. Nanoformulations were centrifuged at 25,000 rpm for 30 minutes at 4 °C using a high-speed cooling ultracentrifuge (Sigma 3-30KS, Sigma Laborzentrifugen GmbH, Osterode am Harz, Germany). After centrifugation, the supernatant was drawn off and analyzed in a UV–visible spectrophotometer (Shimadzu 1800, Kyoto, Japan), with the corresponding *λ*max of the respective active ingredient (357 nm for Ru and 342 nm for Pip). Entrapment efficiency and total drug content were determined using the following formula, according to our previous work (AbouAitah et al., 2020a):

$$\begin{array}{l} \text{EE}\left(\%\right)=\text{initial}\text{amount}\text{of}\text{Ru}\text{or}\text{Pip}\\ \left(\text{theoretically}\text{calculated}\right)\\ –\text{amount}\text{of}\text{free}\text{Ru}\text{or}\text{Pip}\\ \left(\text{actually}\text{measured}\text{amount}\right)\\ \text{in}\text{the}\text{supernatant}/\text{Initial}\text{amount}\text{of}\text{Ru}\text{or}\text{Pip}\\ \left(\text{theoretically}\text{calculated}\right)\times 100. \end{array}$$

(1)




For calculating the loading capacity and total content, we dissolved the 5-mg nanoformulation in 5 mL ethanol, followed by stirring for three hours to forward extraction of the natural agents from the MOF nanoformulation. The solution was then filtered through an Axiva syringe filter (0.2 µm) to exclude MOF particles. For determining Ru or Pip concentration in the samples, we used UV–visible spectrophotometry with the corresponding *λ*max of each natural agent. We calculated the percentage loading capacity and total content as follows:

Totalloadingcontent(%)=amountofRuorPipentrapped/totalweightofMOFcarrier×100

(2)


Totalloadingcapacity(%)=experimentalRuorPipcontent/theoreticalcontentforeach×100

(3)




### *In vitro* release studies

The releasing properties of the nanoformulations were evaluated using a modified dialysis bag diffusion technique according to our previous work on Pip (AbouAitah et al., 2020a). Briefly, the weighed amount of each nanoformulation (5 mg) was transferred to a cellulose dialysis bag (Sigma-Aldrich CHEMIE GmbH, Taufkirchen, Germany) containing 5 mL PBS buffer as the release medium. After the bag was sealed, it was immersed in a glass bottle filled with 50 mL of PBS (pH 7.4), and the bottle was closed. Nanoformulations prepared based on non-modified or silane-modified MOFs were placed in a constant-temperature (37 °C) shaking incubator (GFL 3032, Gesellschaft für labortechnik GmbH, Burgwedel, Germany) at 150 rpm. At specified intervals (1, 2, 3, 4, 5, 6, 8, 12, 24, 36, and 48 hours), we collected a 0.5-mL aliquot of release medium and replaced it with fresh buffer in an equal volume. Before measurements were taken, the solutions were passed through a 0.45-mm Millipore filter. The average cumulative percent of released drug from each nanoformulation was analyzed in triplicate via spectrophotometry. The filtered solutions containing Ru and Pip were measured using a UV–visible spectrophotometer. Co-delivery nanoformulations containing both Ru and Pip were measured once for Ru and once for Pip to characterize the release for each agent from the co-delivery nanoformulations. To analyze the kinetics of the release, we used KineDS3 software (developed at Jagiellonian University, Krakow, Poland), fitting the data to different kinetic models using non-linear and linear regressions.

### *In vivo* pharmacodynamic studies

#### Anti-inflammatory experiment

For the animal studies, we purchased male albino Wistar rats (∼250 ± 50 g; National Research Centre, Giza, Egypt). Animal studies were conducted in keeping with the ethical standards of the pharmacology unit and received approval from the ethics committee of the National Research Centre. Thus, no ethical approval was obtained for the current work. Animals were allocated into 12 groups (Table 1) of eight rats each. Results were calculated as mean values ± SD.

**Table 1.** Animal groups for *in vivo* anti-inflammatory screening.

Group	Classification	Treatment
C	Control	Normal saline
Ref1	Reference (Ref1)	Pip suspended in PBS
Ref2	Reference (Ref2)	Ru suspended in PBS
Ref3	Ref1+Ref2	Mixture of Pip and Ru in PBS
STD	Standard	Diclofenac
G1	MOFs and nanoformulations	ZrMOF
G2		ZrMOFTS-Ru
G3		ZrMOFTS-Pip
G4		ZrMOFTS-Ru-Pip
G4*		Mixture of ZrMOFTS-Ru and ZrMOFTS-Pip
G5		TiMOF
G6		TiMOFTS-Ru
G7		TiMOFTS-Pip
G8		TiMOFTS-Ru-Pip
G8*		Mixture of TiMOFTS-Ru and TiMOFTS-Pip

#### Carrageenan–kaolin-induced paw edema in rats

A blend was prepared consisting of 20% (w/v) kaolin suspension and 1% (w/v) carrageenan, both in saline (Sigma Aldrich, St. Louis, MO). We followed Sur et al. (2019), with minor changes. To induce inflammation, rats were subcutaneously injected on the plantar side of each right hind paw with 0.2 mL of the mixture suspended in normal saline. As described, rats were allocated randomly into 12 groups of eight animals each (Table 1). Control group (C) animals received normal saline at 3 mL/kg of body weight by intraperitoneal injection. Animals in the standard (STD) group received an injection of diclofenac (Novartis, Rueil-Malmaison, France), an anti-inflammatory, at 100 mg/kg of body weight, injected intraperitoneally. The three reference groups (Ref1, Ref2, and Ref3 (mixture of Ref1 and Ref2)) were treated by injection of Ru or Pip or mixture of Ru and Pip intraperitoneally at 100 mg/kg of body weight. Test groups (G1–G8, G4*, and G8*) were injected with different MOF formulations, as shown in Table 1.

Rats were pretreated with different groups for 30 minutes before administration of the carrageenan/kaolin mixture in a single dose. We used a plethysmometer (Panlab, Cornellà de Llobregat, Spain) to measure paw diameter before the stimulus was injected (zero time) and at 1, 2, 3, 4, 5, 6, 8, and 12 hours after the injection, then after 24, 36, and 48 hours. Readings are reported as average variation in paw volume (mL), calculated based on change from the basal value. Results were expressed as the mean percentage of edema inhibition, calculated as follows (Ojewole, 2006):

(4) %edemainhibition=EdemaincreaseinControl–[(edemaincreaseintestgroup)/edemaincreaseinControl]×100

### Leukocyte migration assay (Azza & Oudghiri, 2015)

Subcutaneous air pouches (20 mL sterile air) were formed on the dorsal thorax of all groups (*n* = 8), as described by Haqqi et al. (1999), with some changes. Three days later, 0.5 mL of the carrageenan/kaolin suspension was injected into the resulting cavity in rats of all groups except for control animals in group C, who were injected with 0.9% w/v NaCl. Rats in Ref1, Ref2, and Ref3 were administered each of the pure natural agents intraperitoneally at a total of 100 mg/kg of body weight, and the test groups respectively received intraperitoneal injections of the formulations given in Table 1. Control group C was administered normal saline intraperitoneally at 3 mL/kg of body weight, and the STD group received intraperitoneal diclofenac at 100 mg/kg of body weight. All treatments and control solutions were administered in one dose. At each planned timepoint (at each hour of the first six hours then at 8, 12, 24, 36, and 48 hours), for each animal we injected 5 mL of ice-cold saline solution (0.9%w/v NaCl) into each formed cavity, then collected a sample for counting leukocytes.

### *In vivo* evaluation of antioxidant activity

#### Experimental design and animal exposures

Rats (male albino Wistar, 200 ± 50 g) were kept in plastic cages (six rats/cage) at RT, with access to standard diet and water. Animals were randomly divided into groups as described for the anti-inflammatory experiments, with the following modification: group C (negative control) consisted of untreated rats receiving distilled water for 21 days, and group STD received 100 mg/kg of vitamin C for 21 days. All other groups received a dose of 100 mg/kg of the indicated formulations for each group for 21 days.

All doses were administered intraperitoneally as a single dose after suspension in distilled water at 1 mL/100 g of body weight. At each time interval, we used diethyl ether to anesthetize animals intended for sampling. For obtaining blood samples, we created a retro-orbital puncture and collected the blood into heparinized tubes, which were centrifuged at 4 °C for 15 minutes at 15,000 rpm to separate plasma. The resulting plasma was stored at −20 °C for use in the reducing power and DPPH (2,2-diphenyl-1-picrylhydrazyl) assays (Merghem et al., 2019).

#### Plasma antioxidant capacity using DPPH radical determination

We followed Hasani et al. (2007), with a few modifications, to evaluate the ability of the sampled plasma to scavenge DPPH radicals. In brief, a total of 50 μL of plasma were placed in 1250 μL of a solution of DPPH in methanol (2.4 mg/100 mL methanol), followed by incubation in the dark for 30 minutes. After centrifugation and spectrophotometric analysis, we calculated the plasma antioxidant capacity as follows:

Radicalscavengingactivity(%)=[(Ablank–Asample)/Ablank]×100

(5)




#### Plasma reducing assessed as ferric-reducing antioxidant power

To determine the reducing power of the plasma samples, we followed Narayanaswamy & Balakrishnan (2011) to assay sample antioxidant abilities through formation of a colored complex with potassium ferricyanide (ferric-reducing antioxidant power, or FRAP). One milliliter of plasma was mixed with 0.5 mL each of potassium ferricyanide (1% w/v) and phosphate buffer (0.2 M, pH 6.6), followed by an incubation for 20 minutes at 50 °C. The reaction was terminated by addition of trichloroacetic acid (10% w/v), followed by a 10-minute centrifugation at 3000 rpm. Distilled water and 0.1 mL FeCl3 (0.1% w/v) were used to dilute 0.5 mL of the supernatant. Five minutes later, samples were then analyzed spectrophotometrically, with a higher absorbance indicating greater reducing power.

### Statistical analysis

We analyzed the data using SPSS (Chicago, IL) and give results as means (±standard deviation (SD)) in all tables and figures related to *in vitro* release. For drug-loading content and EE, the data were analyzed with one-way analysis of variance (ANOVA; *p*<.05 with least significant differences). We used Student’s *t*-test or, where more than two groups were compared, one-way ANOVA to compare differences between or among groups in the *in vivo* portions of the study (statistical significance set at *p*<.05).

## Results and discussion

### Post synthetic modification of ZrMOF and TiMOF materials

Figure 1 shows the proposed interaction between ZrMOF and TiMOF materials and TS silane. It is suggested that ZrMOF (with the free carboxylic group) does not react with silane by covalent bonding, whereas TiMOF does react. TiMOF can react with TS silane through the free amino group, which interacts with TS, forming NH bonds. Consequently, silicone (Si) content was greater in TiMOF (2.64 ± 0.25%) than in ZrMOF (0.14 ± 0.01%) (Table S1).

### SEM observations

**Figure 2.**
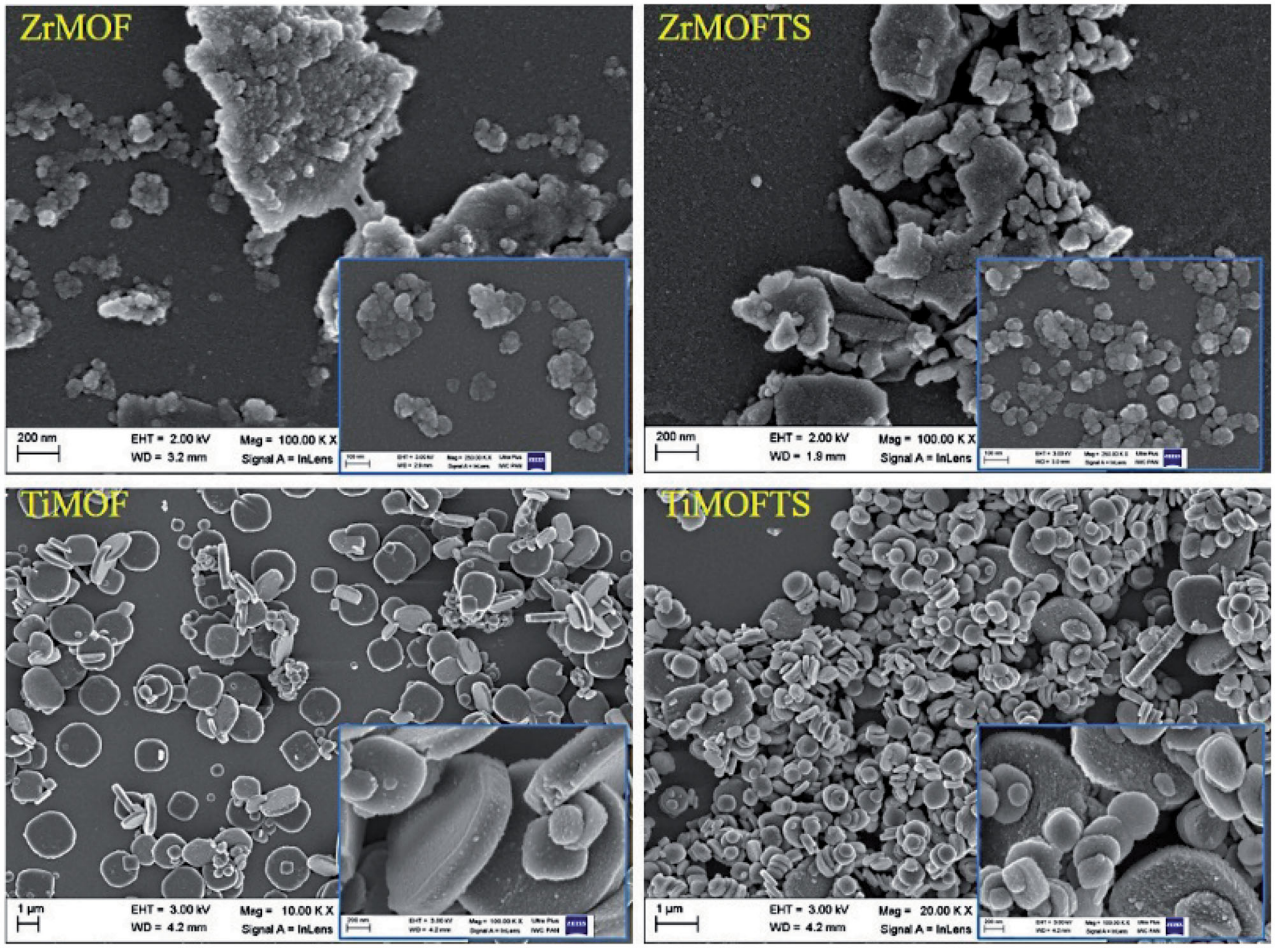
Images of ZrMOF, ZrMOFTS, TiMOF, and TiMOFTS particles, taken using FE-SEM.

According to the FE-SEM images in Figure 2, ZrMOF particles were aggregated with non-uniform structures of a spherical or oval shape. Sizes ranged from nanometers to micrometers. Further surface modification by silane TS groups in ZrMOFTS yielded no differences. Regarding TiMOF, these particles showed a dispersed and uniform structure and were mostly characterized by cubic and hexagonal shapes. We noted no changes for the morphological structures after silane TS group attachment. From a morphological structure perspective, TiMOF seemed to be a more promising drug carrier than ZrMOF.

### XRD characterization

**Figure 3.**
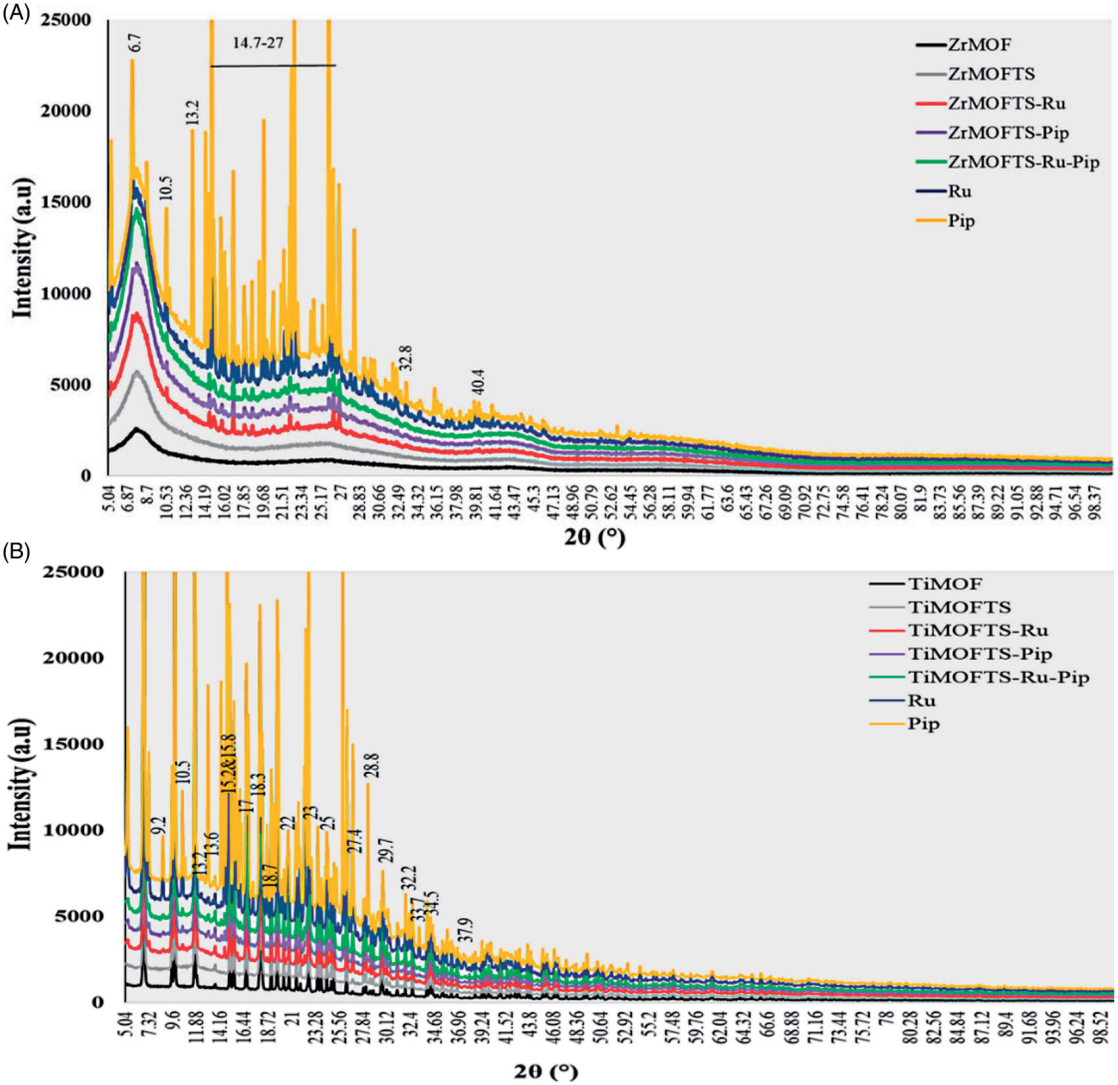
XRD patterns for materials, nanoformulations, and natural agents. XRD patterns for ZrMOF-based preparations (A) and TiMOF-based preparations (B). The analysis was performed on dried powder from prepared materials. 2*θ* from 5° to 100°.

Figure 3(A) shows that all ZrMOF materials exhibited sharp reflection peaks, appearing at low angles (2*θ* = 7.5 and 25.11). The acquisition of these peaks indicates successful preparation of ZrMOF (Yang et al., 2013; Feng et al., 2018; Hassabo et al., 2019). After the surface modification with TS silane groups, we observed no new peaks in the ZrMOFTS pattern. In the nanoformulation patterns, several new diffraction peaks were detected at 6.7, 10.5, 13.2, 14–27, 32.8, and 40.4°, and other small peaks were seen in all nanoformulations (ZrMOFTS-Pip, ZrMOFTS-Pip, and ZrMOFTS-Ru-Pip, corresponding to free Ru or Pip or Ru + Pip). Concerning the TiMOFs (Figure 3(B)), their pattern was characterized by several sharp reflection peaks from low to medium angles (2*θ* = 6.8° to 35°), indicating the successful synthesis of titanium-based MOF. No peaks were observed as a result of the surface modification with TS groups. For nanoformulations, some new diffraction peaks were observed at 9.2°, 10.5°, 13.2°, and 33.7° in all TiMOFTS-Ru, TiMOFTS-Pip, and TiMOFTS-Ru-Pip. In addition, several extensive peaks appeared at the same positions because of overlapping peaks of the drugs and TiMOF. These peaks indicate the presence of Ru or Pip. As indicated by XRD results for nanoformulations, the Ru and Pip mostly loaded into the MOFs, and small fractions of the drug molecules could be found on the surface in the crystalline phase. This observation confirms that loading of the natural agents into the nanoformulations either singly or combined was successful, in line with previous reports of MOFs loaded with various drugs (Rezaei et al., 2018; Pham et al., 2020).

### FTIR characterization

**Figure 4.**
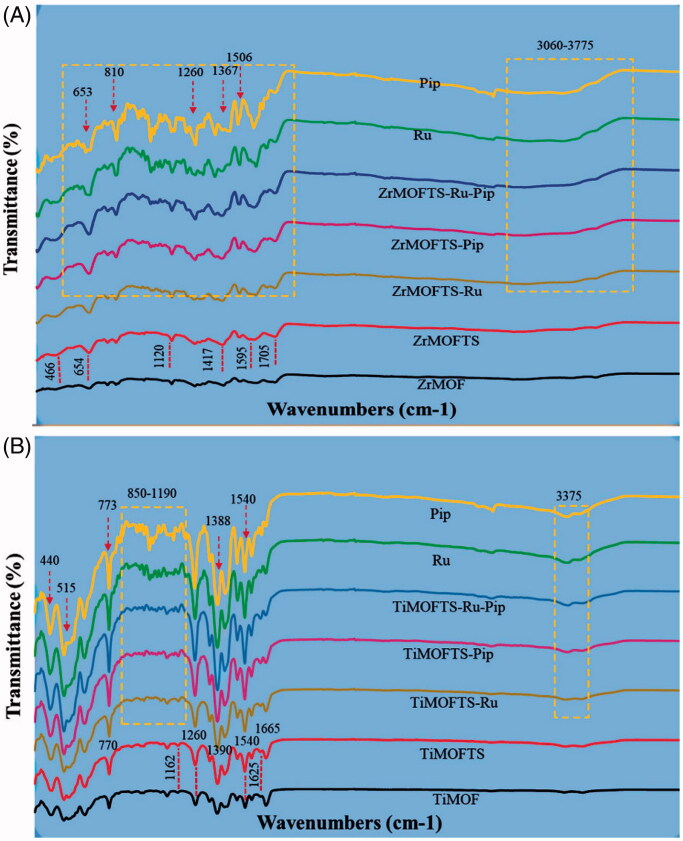
The FTIR spectra of MOF materials, nanoformulations, and free natural agents. ZrMOF-based preparations (A) and TiMOF-based preparations (B). The measurements were performed for powders using an ATR unit of the FTIR device from 400 to 4000 cm^−1^ at RT.

As shown in Figure 4, several peaks could be seen between 400 and 1750 cm^−1^, confirming the similar surface compositions for ZrMO and TiMO (Vilela et al., 2017; Sarker & Jhung, 2019; Li et al., 2020). Moreover, spectra obtained for pure prodrugs Pip and Ru presents main IR bands in the same spectral range 400–1750 cm^−1^. Therefore, the comparison of the spectra obtained for the samples before and after modification is difficult. However as shown in Figure 4(A), in the ZrMOFTS spectrum, several bands (654, 1120, and 1705 cm^−1^) are slightly more intense compared to the bands for ZrMOF. The peaks at 654 cm^−1^ and 1120 cm^−1^ especially may reflect vibrations from stretching of the silane TS groups’ Si–O–Si and Si–O bonds (Mahdavi et al., 2015). Other highlighted peaks suggested the presence of ethoxy groups in modified materials (Kim et al., 2009). Taken together, these results point to the successful surface functionalization of TS silane groups into/onto MOFs. For two types of nanoformulations, new band corresponding to Ru and Pip were detected in 1130 cm^−1^. For samples with Pip very weak peak related to medicine was detected at 2940 cm^−1^. In addition, peaks overlapped with those related to ZrMOs at 653, 810, 1260, 1367, and 1506 cm^−1^ are present. As shown in Figure 4(B), in the TiMOFTS spectrum, peaks at 400–650 cm^−1^ were shifted, whereas bands at 770, 1160, 1540, and 1625 cm^−1^ had higher intensities compared to TiMOF. This suggests that the TS silane groups were attached in TiMOFs. For nanoformulations, new bands were seen in the 850–1190 cm^−1^ spectral range, pertaining to Ru or Pip or their combination. Also, increased intensities were detected at 440, 515, 773, 1388, and 1540 cm^−1^ for nanoformulations compared to TiMOF and TiMOFTS. It suggests presence of Ru and/or Pip in the nanoformulations.

The FTIR results indicate successful incorporation of Ru and/or Pip into the materials. These results are consistent with previous reports describing other drug loading into MOFs (Rezaei et al., 2018; Chen et al., 2019; Liu et al., 2019). As indicated by the collective results from FTIR and XRD, Ru and/or Pip were mainly loaded into the MOFs, with some fraction of molecules remaining on the surface in a crystalline state.

### Thermal analysis

#### STA characterization

**Figure 5.**
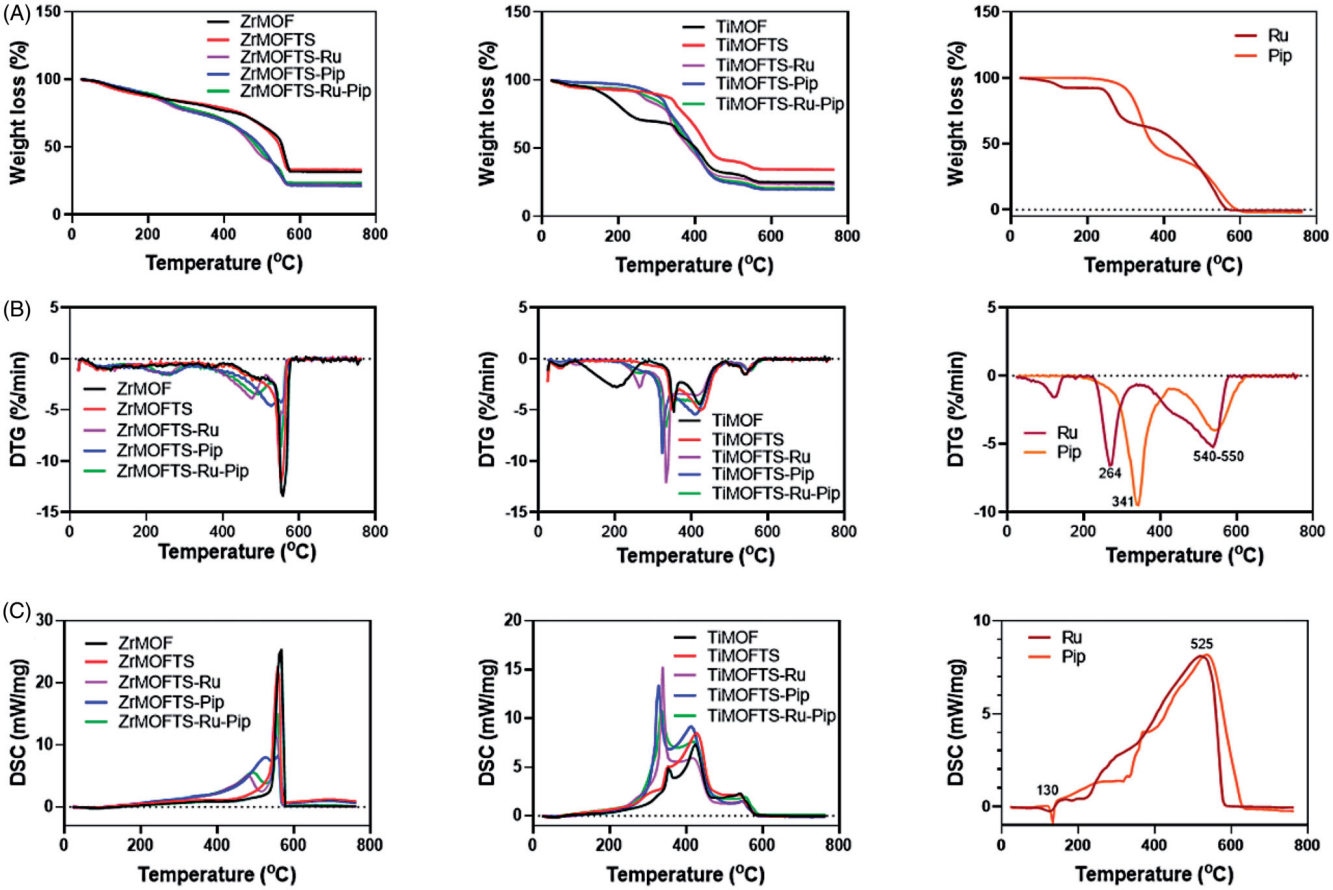
Characterization of MOFs, nanoformulations, and free natural agents by STA coupled with DSC. Weight loss measurements by STA for MOFs, nanoformulations, and free natural agents (A); DSC curves for MOFs, nanoformulations, and free natural agents (B); and DTG thermograms for MOFs, nanoformulations, and free natural agents (C).

Figure 5 and Table 2 show the results of the thermal analysis of the materials prepared at all stages. Thermogravimetry data indicate that in the experimental temperature range, the weight loss varied according to type of MOF material and reached about 68 wt.% and 75 wt.% for ZrMOF and TiMOF, respectively (Figure 5(A)). These results are consistent with data concerning mass loss obtained for MOF materials, including Zr-MOF (Santos et al., 2020).

**Table 2.** Thermogravimetric analysis, preparation conditions, and drug-loading properties of Ru and Pip in nanoformulations using the UV–vis method.

Formula	Preparation conditions			Weight loss, wt.%*	Drug-loading measurements by UV–vis	
	Drug:MOF ratio	Volume/solvent	Temperature/stirring speed		Mean TLC (% ±SD)	Mean EE (% ±SD)
ZrMOF				67.9		
ZrMOFTS				66.0		
ZrMOFTS-Ru	1:2	15 mL/ethanol	RT/200 rpm	77.7	11.97 ± 0.65^c^	40.13 ± 2.33^f^
ZrMOFTS-Pip	1:2	15 mL/ethanol	RT/200 rpm	78.5	12.71 ± 1.97^c^	34.58 ± 2.77^f^
ZrMOFTS-Ru-Pip	0.5 Ru:0.5 Pip (total 1):2	15 mL/ethanol	RT/200 rpm	67.6	As Ru: 9.34 ± 1.04^e^	73.54 ± 2.85^a^
					As Pip: 9.08 ± 0.66^e^	69.48 ± 1.46^b^
TiMOF				75.2		
TiMOFTS				65.7		
TiMOFTS-Ru	1:2	15 mL/ethanol	RT/200 rpm	76.5	15.56 ± 1.24^b^	53.67 ± 1.65^d^
TiMOFTS-Pip	1:2	15 mL/ethanol	RT/200 rpm	80.1	17.11 ± 1.43^a^	50.25 ± 2.23^e^
TiMOFTS-Ru-Pip	0.5 Ru:0.5 Pip (total 1):2	15 mL/ethanol	RT/200 rpm	79.3	As Ru: 13.69 ± 0.96^c^	65.29 ± 3.82^c^
					As Pip: 12.77 ± 0.99^c^	63.48 ± 1.96^c^

The superscript letters (a, b, c, etc.) indicate significant differences by ANOVA through least significant differences between groups at *p*<.05. Different letters indicate significant differences, and the same letters indicate no significant differences.

* By UV–Vis analysis.

After surface modification with TS silane groups, there was a gain noted in both materials ZrMOFTS and TiMOFTS. This change could be attributable to the different extent of silane modification, affecting the Si oxidation and/or changes in thermal stability of the silane groups (Sarker & Jhung, 2019). This behavior was in accordance with previous work (Li et al., 2014).

The DTG patterns (Figure 5(B)) of the modified MOFs were characterized by three stages of mass loss associated with adsorbed water removal (centered at ∼90 °C), decomposition of the organic content (centered at ∼220 °C), and destruction of MOF structure (centered at ∼580 °C for ZrMOF and 420 °C for TiMOF) (Sarker et al., 2019). Apart from modified materials, in nanoformulations, there was an increment in weight loss observed, verifying the success of loading to both MOFs. As expected, pure Ru and Pip were totally decomposed (almost 100 wt.%). All DTG curves (Figure 5(B)) for nanoformulations showed intensification compared to DTG curves of modified MOFs, as a result of the higher weight loss (Table 2). There were two stages of mass change during the heating to 800 °C. The first stage resulted in peaks shifted at ∼230 °C and ∼320 °C, corresponding to the main peaks detected for free Ru and Pip at 264 °C and 341 °C, respectively. The second stage showed peaks shifted from center at ∼540–550 °C for Ru and Pip, respectively. This shift is connected to the decomposition/volatilization of both natural agents used. Of note, the shifted peaks in the nanoformulations appeared to correspond to those for free Ru and Pip, confirming the successful loading process for either single or double drug loading (Cunha et al., 2013a,b; Sarker & Jhung, 2019; Sarker et al., 2019).

#### DSC characterization of materials

The DSC patterns (Figure 5(C)) of all materials during the experiments indicated that the exothermic process correlated with mass loss. However for Pip and Ru, an endothermic signal was detected below 200 °C, probably corresponding to melting process. Prior to surface modification, a sharp exothermic peak centered at ∼570 °C was detected, a feature unique to ZrMOFs. After the surface modification, we observed the same peak at a lower intensity. Through preparation of the nanoformulations, the DCS curves of Zr-MOFTS-Ru, ZrMOFTS-Pip, and ZrMOFTS-Ru-Pip showed new exothermic peaks at 473–524 °C, corresponding to free Ru and Pip. The free Ru and Pip presented broad exothermic peaks centered at ∼525 °C, arising from their decomposition. These peaks confirmed the presence of natural agents in the nanoformulations.

Concerning the TiMOF material, two broad peaks characteristics for TiMOF were detected at 355 °C and 426 °C. These peaks were shifted and had a little higher intensities compared to pristine TiMOF, indicating the attachment of silane groups. The nanoformulations resulted in new sharp peaks centered at about 325 °C, which could be shifted from the original peaks for the natural agents. Other peaks appeared at the same positions or only slightly shifted from free Ru and Pip. These peaks indicate the successful loading of pro-drugs into the nanoformulations. As can be seen, the DSC changes for all of the nanoformulations correlate with the DTG data.

### Measurements of zeta potential

**Figure 6.**
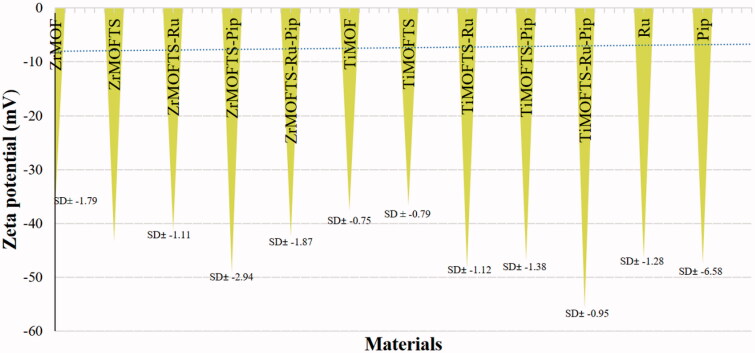
The zeta potential of MOFs, nanoformulations, and free natural agents after suspension in deionized water (pH adjusted to 7.4 for all measurements at RT).

Zeta potential is crucial for estimating the surface charge of nanoparticles to understand their stability in suspension. All pristine MOFs, TS silane-modified MOFs, and nanoformulations were measured based on their suspension in deionized water. We also measured free Ru and Pip for comparison. As shown in Figure 6, all materials displayed negative zeta potential values of around −37 to −55 mV. Among the ZrMOFs, the lowest value was obtained for ZrMOF (–37.11± −1.8), whereas the highest values were recorded for the ZrMOFTS-Pip nanoformulation (–49.01± −2.94). Similarly, TiMOF and TiMOFTS had the lowest negative zeta values (–37.56± −0.75 and −36.51± −0.79, respectively), whereas the highest value was detected for the TiMOFTS-Ru-Pip nanoformulation (–55.53± −0.95). Additionally, free Pip and Ru had similar negative zeta values of −47.35± −6.58 and −46.36± −1.28, respectively. For ZrMOF, the surface modification altered the zeta potential from −37.1 (ZrMOF) to −43.21 (ZrMOFTS), in good agreement with previous results for MOFs (Hidalgo et al., 2017; Li et al., 2020). These findings may indicate that all of these materials are electrically stable when suspended in water. One plausible reason is the high negative/positive zeta potential values that generate repulsion between adjacent particles in solution, resulting in good stability and limiting aggregation (Frank et al., 2020). Generally, sufficient repulsive force is indicated by a zeta potential value ranging from >–30 mV to +30 mV, leading to better physical stability (Joseph & Singhvi, 2019). In this context, an emulsion with zeta potential values ranging from −41 to −50 mV indicates good stability (Losso et al., 2005). Accordingly, our prepared system, especially nanoformulations with Ru or Pip, could be more stable than others.

### Particle size measurement

Table 3 shows the mean particle size of nanoformulations by means of DLS measurements. The results indicate that the Zr-based nanoformulations had larger particle sizes when compared to Ti-based nanoformulations. In addition, the dual loading affected the particle size, with increases detected for nanoformulations consisting of both Ru and Pip compared to single loading. The same effect was obtained for the mean PDI. The ZrMOFTS-Ru-Pip nanoformulation had the highest PDI, almost within the micro range, mainly because of the high-molecular-weight zirconium as the inorganic moiety, the high-molecular-weight carboxyl branching, and the involvement of both Ru and Pip in the same formulation. Furthermore, the PDI of all formulations came within a range that should assure their stability. Of note, the results of PDI were in agreement with those for zeta potential, which reflected exceptionally stable formulations.

**Table 3.** Particle size measurements by DLS of prepared nanoformulations in deionized water.

Nanoformulation	Mean particle size (nm ± SD)	PDI (±SD)
ZrMOFTS-Ru	465.80 ± 18.34	0.374 ± 0.091
ZrMOFTS-Pip	552.30 ± 13.01	0.361 ± 0.024
ZrMOFTS-Ru-Pip	928.91 ± 31.52	0.522 ± 0.031
TiMOFTS-Ru	234.20 ± 9.57	0.229 ± 0.041
TiMOFTS-Pip	229.00 ± 5.92	0.213 ± 0.055
TiMOFTS-Ru-Pip	656.75 ± 27.70	0.302 ± 0.021

### Drug-loading properties

In the present study, Ru and Pip independently or combined loaded to Zr-based or Ti-based MOFs. All formulations were subjected to the same preparation method, using the same weight ratios (drug:MOFs) among the preparation components. As Table 2 shows, total loading capacity (TLC) did not differ significantly for single versus dual loading of Ru and/or Pip into the nanoformulations (*p* ˂ .05). We also found no significant difference in EE with single loading of Ru or Pip but did find differences for EE when both Ru and Pip were loaded together in nanoformulations. For TiMOF-based nanoformulations, the results showed a significant effect on TLC, but no significant differences in EE between nanoformulations. As can be seen, TI-MOF-based nanoformulations significantly increased TLC for Ru and/or Pip compared to ZrMOF-based nanoformulations. Additionally, the EE for Ru or Pip significantly increased with Ti-MOF compared with Zr-MOF nanoformulations when used in single loading. In contrast, only Zr-MOF nanoformulations significantly increased EE of Ru and Pip loaded in combination compared to TiMOF material. The TiMOFTS-Pip nanoformulation had the maximum TLC for Pip (17.11 ± 1.43%), and the TiMOFTS-Ru nanoformulation had the maximum for Ru (15.56 ± 1.24%). The obtained TLCs for Ru and Pip are in line with previous reports for drugs loaded to various MOFs, such as DOX (∼16 wt.%; Bi et al., 2018) and gentamicin (19 wt.%; Soltani et al., 2018). In general, both TLC and EE were significantly affected by type of MOF material.

**Figure 7.**
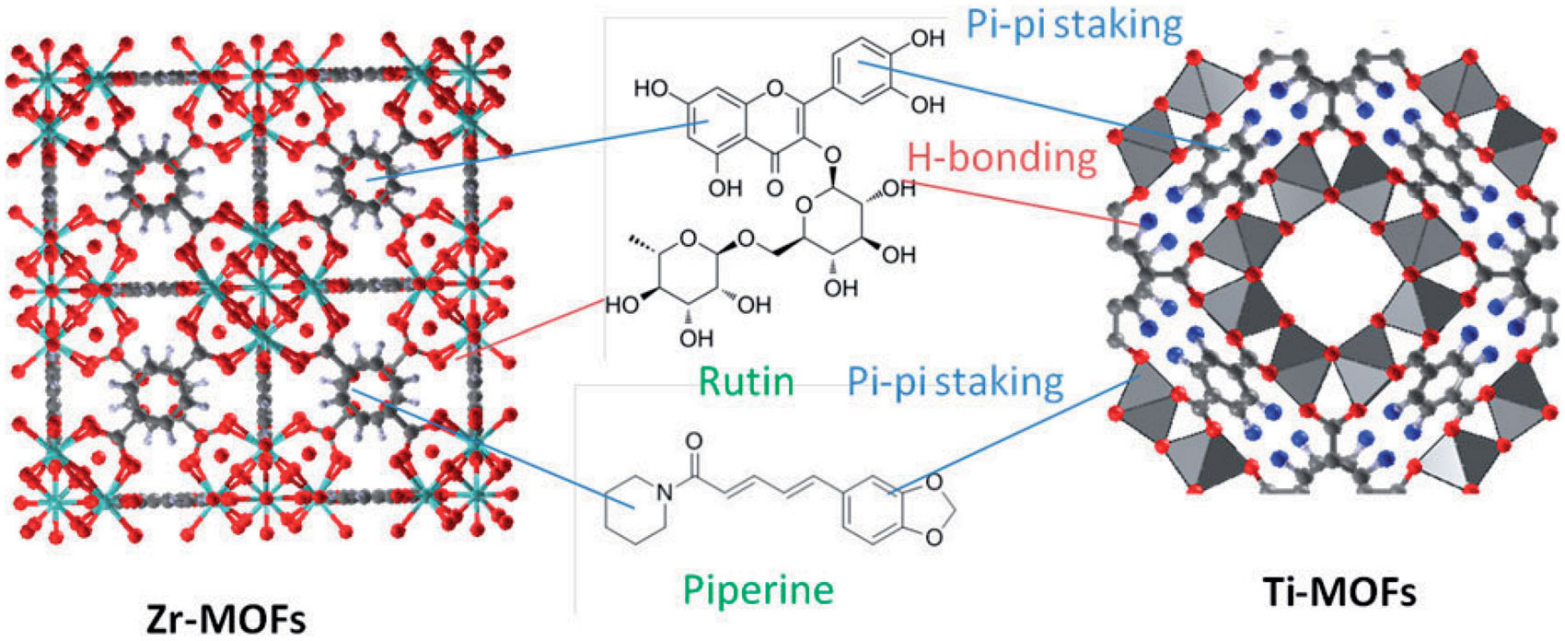
The suggested incorporation mechanism of drug onto MOFs materials.

Metal organic frameworks are excellent drug carrier due to the synergy of the effect of pores inside the framework and interaction with functional groups like amine and carboxylate groups. In the studied case, Ru and Pip can be loaded onto/into MOFs materials taking advantage of: (i) hydrogen bonding with free amino and carboxylate group, (ii) chemical bonding with free metal ion center (silicon) leading to form Si–O bond, and (iii) physical adsorption into pores of the framework via pi–pi staking (Figure 7).

### *In vitro* release kinetics

The release from non-modified MOFs (ZrMOF-Ru, ZrMOF-Pip, TiMOF-Ru, and TiMOF-Pip) at pH 7.4 (Table S2), resulted in fast release profiles, taking place within 24 hours. The release kinetics in Ti-based formulations showed a significant difference in mean release efficiency (MRE) value compared to their Zr analogues. The results suggest that the metal component of the nano carrier system might be the limiting factor in controlling the release profile of both Ru and Pip in nanoformulations.

**Figure 8.**
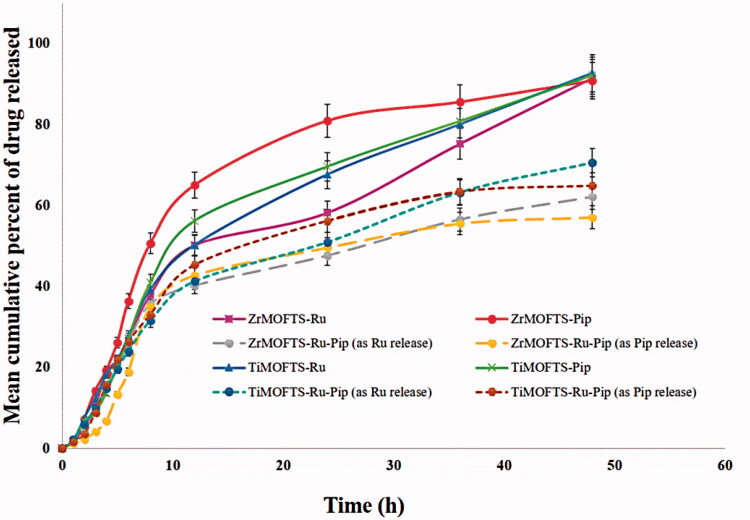
*In vitro* release of Pip or Ru from nanoformulations prepared depending on silane-modified MOFs materials (ZrMOFTS or TiMOFTS). The release tests were done in PBS buffer medium. In case of co-delivery nanoformulations containing Ru and Pip, the release was measured by UV–vis and calculated once for each of them from the release solutions, as shown in dotted lines. Data are mean ± SD.

Figure 8 displays the cumulative release of Ru or Pip from nanoformulations as a function of time from nanoformulations designed using silane-modified MOFs. As shown in Figure 8, at 48 hours, Ru or Pip nanoformulations had a cumulative release of >90%. For dual-delivery nanoformulations (ZrMOFTS-Ru-Pip and TiMOFTS-Ru-Pip), we calculated the release of each natural agent. Their release profiles (dotted lines) indicated that within 48 hours, ∼62% and ∼56% as Ru and Pip, respectively, released from the ZrMOFTS-Ru-Pip formulation and ∼71% and ∼65%, respectively, released from the TiMOFTS-Ru-Pip formulation. It is seen also that the release profiles are as follows. Zr-MOFTS-Pip showed a fast release, probably due to lack of chemical connection between the framework and drug structure. In contrast, Ti-MOFTS-Pip showed a slow release, probably due to chemical bonding between silicone metal center and drug. This effect can be used to control of the release behavior of drugs and for constructing novel drug carriers. Another feature of release from both MOF materials was that all release profiles could be described by two stages: a zero-order release effect as the first stage, within 12 hours, and a stable sustained release as the second stage, from 12 hours to the end of the experiment. As such, mixed release patterns are likely the result of having no burst in the first stage, with a slight increase in Ru or Pip release at the second stage. The observation of a two-stage pattern is in agreement with previous reports, including release of ibuprofen from various MOFs (Silva et al., 2016; Rojas et al., 2018; Sarker et al., 2019; Pham et al., 2020), 5-fluorouracil from Mg-MOFs (Hu et al., 2020), caffeine from ZrMOFs (Sarker & Jhung, 2019), and DOX from a zeolitic imidazolate MOF (Bi et al., 2018).

Next, we fitted the release profiles of Ru and Pip obtained from both types of MOFs to the following kinetic models: zero-order, first-order, Hixson–Crowell, Korsmeyer–Peppas, and Higuchi. With linear regression modeling only, the results indicated that Ru and Pip were released from nanoformulations according to zero-order kinetics (*R*^2^=0.98–0.99). On the other hand, investigation of the linear and non-linear regressions together showed that Korsmeyer–Peppas had the best fit (*R*^2^=0.99–1.00) (Table 4). Thus, the *in vitro* release of both Ru and Pip followed the same kinetics regardless of metal composition (Zr or Ti) within the nanocarrier MOFs. These results are consonant with those of earlier studies of the *in vitro* kinetics of various drug material release from different MOF structures (Li et al., 2019a; Santos et al., 2020). Zero-order kinetics describes the release kinetics for drug diffusion from reservoir-based systems including MOFs, based on Fickian diffusion (Horcajada et al., 2008; Peppas & Narasimhan, 2014; Pham et al., 2020). Consequently, the zero-release up to about 12 hours demonstrates that MOF structures can efficiently control drug release without a premature lag or burst. Generally, the Korsmeyer–Peppas model is used to describe the surface degradation/erosion of a formulation containing the drug (Costa & Sousa Lobo, 2001; Rothstein et al., 2009). With surface erosion, degradation is restricted to the outermost surface of the porous system without affecting the interior (Pham et al., 2020). Comparing the release of Ru and Pip from both MOFs showed no significant differences for the mean cumulative release (MCR) release parameter. Thus, the MOFs did not affect the maximum released amount of these agents regardless of type. Concerning the MRE kinetic parameter, Ru and Pip from the Zr-MOF nanoformulations differed significantly but did not differ significantly in the Ti-MOF nanoformulations. Significant differences were observed in the other two kinetic parameters: mean release rate (MRR) and mean release time (MRT), the mean time required for maximum release of a drug or medical agent from its carrier system or dosage form. MRT is a kinetic parameter that is a function of either MRR or MRE or both. As MRT increases, both MRE and MRR are expected to decrease, unless influenced by other external factors. A decrease in MRT reflects a highly efficient system that allows easy release of a drug into the medium and a high degree of solubility, as indicated by a high MRR value. Furthermore, for loading of the Ru and Pip in combination, results showed that Ru kinetically exceeded Pip, given the highly significant difference between them for MCRP, MRR, and MRT. The amount of natural agent that is loaded directly affects the relation between MRT on one side and MRR and MRE on the other. Overall, Ti-based nanoformulations proved to be more efficient as nanocarrier systems for Ru and Pip singly or together compared to the Zr-based nanoformulations. A review of the literature shows that the release kinetics of Ru can vary depending on the drug carrier used in the various nanosystems but that it mostly releases with mixed kinetic mechanisms, similar to our results. In this way, the release pattern of Ru from solid lipid nanoparticles is a good fit to the first-order and Korsmeyer–Peppas models (Pandian et al., 2020), in keeping with results showing Eudragit nanosphere release with Korsmeyer–Peppas and phase II kinetics (Asfour & Mohsen, 2018), and mesoporous silica nanoparticle release through Higuchi and first-order kinetics (Karnopp et al., 2020). Concerning the co-delivery strategy, the release of Ru or Pip with other drugs also can occur via combined kinetic mechanisms. The release of Pip and DOX follows the kinetics of Korsmeyer–Peppas and *n*-value, suggesting a mechanism of Fickian’s diffusion from lecithin-chitosan nanoparticles (Alkholief, 2019). The release kinetics for Ru and curcumin co-delivery, however, showed a non-Fickian transport model from chitosan nanoparticles (Ramaswamy et al., 2017).

**Table 4.** *In vitro* kinetic release of Ru and Pip at pH 7.4 from the nanoformulations.

Kinetic model and release parameters	Nanoformulation							
	F1, ZrMOFTS-Ru	F2, ZrMOFTS-Pip	F3, ZrMOFTS-Ru-Pip (examining Ru release)	F3, ZrMOFTS-Ru-Pip (examining Pip release)	F4, TiMOFTS-Ru	F5, TiMOFTS-Pip	F6, TiMOFTS-Ru-Pip (examining Ru release)	F6, TiMOFTS-Ru-Pip (examining Pip release)
Kinetic model	Korsmeyer–Peppas (power law)							
*R* ^2^	0.99 ± 0.02							
MCRP	91.43 ± 3.59	90.81 ± 5.07	62.08 ± 2.04	56.08 ± 1.10	92.64 ± 4.25	92.06 ± 3.82	70.53 ± 2.12	64.86 ± 1.01
MRE (% ±SD)	57.51 ± 1.00	69.52 ± 2.10	44.56 ± 1.10	43.89 ± 1.53	61.43 ± 1.11	62.90 ± 2.41	48.01 ± 1.00	49.51 ± 2.04
MRR (%/h ± SD)	3.02 ± 0.05	4.12 ± 0.02	2.26 ± 0.01	2.07 ± 0.01	3.88 ± 0.02	3.14 ± 0.01	2.98 ± 0.01	2.35 ± 0.02
MRT (h)	17.81 ± 0.42	11.26 ± 0.41	13.75 ± 0.30	10.01 ± 0.25	16.17 ± 0.13	15.20 ± 0.25	12.33 ± 0.41	9.36 ± 0.09

It is seen that modification with silane of MOFs surface plays a crucial role for *in vitro* release kinetics. This becomes clear when the release profile of silane-free formulations is compared to silane-modified nanoformulations. The silane modification in MOFs leads to a long sustained release effect (within 48 hours) compared to fast/burst release effect (24 hours) for MPFs without silane modification. All surface-modified MOF carriers were shown to be convenient DDS for release of potent drugs, in small or extended doses. They versatility of their design permits tailoring of adequate DDSs, optimized to the desired scope of the suggested treatment plan, as per patient’s medical requirements (Ganesh et al., 2012). Preference is for silane-based MOF rather than the silane free MOF structures.

### *In vivo* studies

The antioxidant and anti-inflammatory activities of Ru and Pip have previously been described (Selvendiran et al., 2003; Bang et al., 2009; Lee et al., 2012; Mahmoud, 2012; Ramaswamy et al., 2017; Enogieru et al., 2018). Here, we evaluated whether these properties can be enhanced using a nanoformulation system. Recent evidence indicates that nanoformulations could be an alternative way to improve the pharmacological effects of natural agents (Yavarpour-Bali et al., 2019). Additionally, the pharmacological effects may offer medical value against many neurodegenerative diseases (Khan et al., 2020), including Alzheimer’s, oxidative stress, Parkinson’s, and Huntington’s, possibly because of a shared underlying mechanism of neuronal loss, inflammation, and oxidative stress (Enogieru et al., 2018). Targeted therapies for these conditions are crucially needed because of the progressive neuronal loss and related impairments in cognition and memory (Aarsland et al., 2009; Magalingam et al., 2018).

### Evaluation the nanoformulations for anti-inflammatory effects

#### Amelioration of induced paw edema

**Figure 9.**
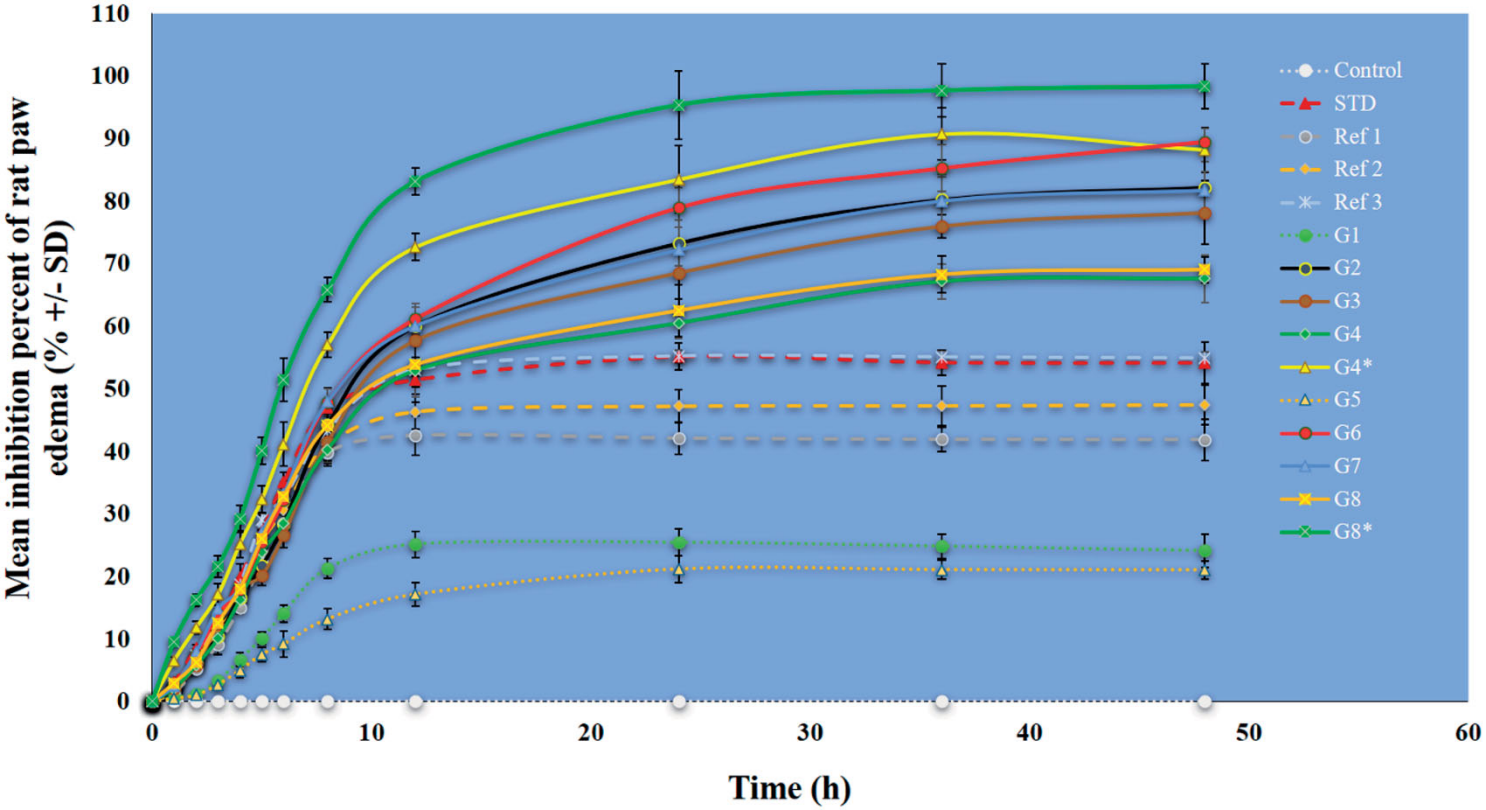
Mean percent inhibition of rat paw edema by various nanoformulations versus free natural agents or standard drug. The treatments were done in a single dose (intraperitoneal injections) in rats. Data are presented as mean ± SD. Dotted lines represent the MOF materials modified with TS, free natural agents, and standard drug. C (Control, normal saline), Ref1 (Pip suspended in PBS), Ref2 (Ru suspended in PBS), Ref3 (mixture of Pip and Ru in PBS), STD (standard diclofenac drug), G1 (ZrMOFTS), G2 (ZrMOFTS-Ru nanoformulation), G3 (ZrMOFTS-Pip nanoformulation), G4 (ZrMOFTS-Ru-Pip nanoformulation), G4* (mixture of ZrMOFTS-Ru and ZrMOFTS-Pip nanoformulation), G5 (TiMOFTS), G6 (TiMOFTS-Ru nanoformulation), G7 (TiMOFTS-Pip nanoformulation), G8 (TiMOFTS-Ru-Pip nanoformulation), and G8* (mixture of TiMOFTS-Ru and TiMOFTS-Pip nanoformulation).

Paw edema induced in rats by carrageenan is a typical phlogistic agent for systemic evaluation of anti-inflammatory activity, and it is still used because of its non-antigenic nature and absence of noticeable adverse reactions (Eze et al., 2019). However, kaolin, because of its clay nature, may be preferential over carrageenan because it does not lead to antigenicity or hypersensitivity reactions. Therefore, the carrageenan and kaolin-induced edema have been combined into one model and widely used (Pashmforosh et al., 2018; Sur et al., 2019). We followed this model here. As shown in Figure 9, the results demonstrated that nanoformulations containing Ru or Pip as single loading caused a significant inhibition in paw edema compared to nanoformulations containing combined loading of Ru and Pip together (G4 and G8). There was a greater percent inhibition with injection of G4* and G8* as a mixture of nanoformulations containing Ru and Pip, respectively. One explanation for this observation is the better anti-inflammatory action obtained for nanoformulations containing one natural agent rather than two natural agents, or a synergistic effect may have occurred. The results generally are in agreement with the *in vitro* release findings (Figure 8) showing that single-agent nanoformulations released more agent than dual-agent nanoformulations. We note that rat paw edema reached a maximum inflammatory volume at three hours after the stimulant injection, which would explain the initially low inhibition percentage of the administered treatment in all groups. The significantly lower inhibition ability of the free Ru and Pip (Ref1 and Ref2) compared to the STD group is mainly attributed to the enhanced solubility of the standard drug. Briefly, these data showed significant inhibition exerted by nanoformulations compared to standard diclofenac drug or free Ru and/or Pip. The findings are in accordance with our previous data showing a notable anti-inflammatory influence in an induced versus free-form rat model for nanoformulation-based mesoporous silica nanoparticles for the flavonoid quercetin and shikimic acid (AbouAitah et al., 2020b). Other studies also have reported this effect in animal tests (Xu et al., 2011; Rachmawati et al., 2016; de Almeida et al., 2018), showing the importance of nanoformulation delivery compared to the traditional methods of delivering anti-inflammatory drugs.

### Leukocyte migration

Inflammation induced by carrageenan/kaolin occurs in two phases. The first phase involves histamine, kinin, and serotonin release, and the second phase involves prostaglandin, protease, and lysosome enzyme release. The first phase proceeds during the first hour after stimulus injection, and the second phase carries over into hours 3 and 4 (Mondal et al., 2019). As shown in Figure 9, nanoformulations significantly reduced the paw volume as compared to the free natural agents, STD, and or control groups. The results also were confirmed through leukocyte count, in which a decrease indicates the bio-efficiency of the injected substance/material through an anti-inflammatory effect. The maximum number of leukocytes migrating to the air pouch after stimulus injection was about 5.3 × 10^5^ cells/mL and was found in control group C. This count was significantly higher than in any other group. The leukocyte count for the STD group was about 2.4 × 10^5^ cells/mL, and the Ref1, Ref2, and Ref3 groups, treated respectively with free Ru, Pip, and their mixture, had significantly lower leukocyte count (respectively 3.4 × 10^5^ cells/mL, 3.1 × 10^5^ cells/mL, and 2.6 × 10^5^ cells/mL) compared to the control and STD groups. Of the reference groups, Ref3 (Ru + Pip) had the lowest value, implying greater efficiency, possibly because of the bioenhancing nature of Pip in the drug combination. As noted in the rat paw edema experiment, diclofenac was rapidly eliminated compared to the extracts, whose effect lasted until the end of the experiment. All nanoformulation groups showed a significant reduction compared to other treatments, in agreement with previous reports (de Almeida et al., 2018). Additionally, groups G4* and G8* showed the best results, with the lowest leukocyte counts at 1.4 × 10^5^ cells/mL and 1.1 × 10^5^ cells/mL, respectively. A possible mechanism of action could be changed in leukocyte migration into tissues and the target organ, in addition to a putative anti-prostaglandin and antioxidant effect of both Ru and Pip.

#### Evaluation of antioxidant effects in rats

Among the most commonly used biomarkers in the assessment of antioxidant effectiveness is plasma antioxidant capacity. The idea is based on the network of a large number of endogenous antioxidants in plasma. These antioxidants can show complementary or synergistic behavior, providing efficient protection against reactive oxygen species. Among the methods available for evaluating antioxidant activity are FRAP, trolox equivalent antioxidant capacity, total radical absorption potential, and the radical scavenging activity of DPPH. In the present work, we used two complementary tests: DPPH scavenging activity and FRAP.

### Effect on plasma antioxidant capacity using DPPH radical and FRAP reducing power

**Figure 10.**
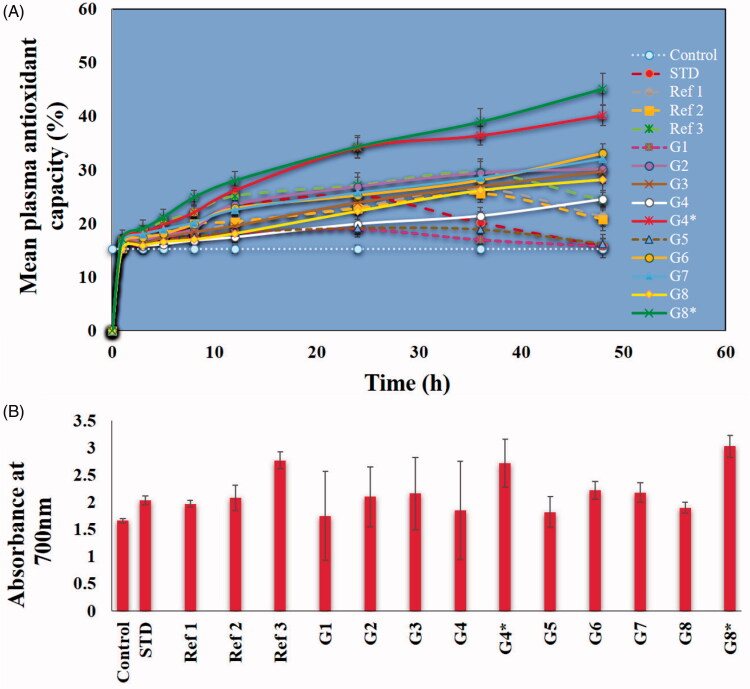
Mean plasma antioxidant capacity of various MOFs versus free natural agents or standard drug. Plasma antioxidant capacity using DPPH radical (A) and FRAP reducing power (B). The treatments were done in a single dose (intraperitoneal injections) in rats. Data are presented as mean ± SD. Dotted lines represent the functionalized MOF materials, free natural agents, and standard drug. C (Control, normal saline), Ref1 (Pip suspended in PBS), Ref2 (Ru suspended in PBS), Ref3 (mixture of Pip and Ru in PBS), STD (standard diclofenac drug), G1 (ZrMOFTS), G2 (ZrMOFTS-Ru nanoformulation), G3 (ZrMOFTS-Pip nanoformulation), G4 (ZrMOFTS-Ru-Pip nanoformulation), G4* (mixture of ZrMOFTS-Ru and ZrMOFTS-Pip nanoformulation), G5 (TiMOFTS), G6 (TiMOFTS-Ru nanoformulation), G7 (TiMOFTS-Pip nanoformulation), G8 (TiMOFTS-Ru-Pip nanoformulation), and G8* (mixture of TiMOFTS-Ru and TiMOFTS-Pip nanoformulation).

Oral administration of Ru and/or Pip either as free or nanoformulations led to a general enhancement of the plasma antioxidant capacity (Figure 10(A,B)). Compared with results in the control group, this increase (15.25 ± 1.46%) was statistically significant, represented as the basal line in the figure. Also, loading of single Ru or Pip was associated with higher antioxidant effects than when the combination was loaded. Administration of G4* (40.20 ± 1.02) and G8* (45.10 ± 2.08) resulted in the highest significant antioxidant effect, compared to all other nanoformulations and standard drug. Thus, administering the two independent single-drug nanoformulations together by mixing them after preparation had a more significant effect than their administration in a co-delivery nanoformulation. The likely explanation is competition between Ru and Pip within the same MOF, as well as their shared antioxidant properties. Similarly, the plasma reducing power based on FRAP analysis indicated that G4* and G8* had the highest and most significant antioxidant effect compared to other groups and control.

We can draw the following conclusions from the antioxidant and anti-inflammatory effects detected here. First, the use of MOF carriers improves these effects compared to free natural agents and standard drugs. These results are in accord with other reports on nanodelivery systems versus free natural agents (de Almeida et al., 2018). Second, a mixture of single-loaded Ru and Pip enhances the antioxidant and anti-inflammatory effects compared with dual-loading versions. Third, the anti-inflammatory and antioxidant effects of Ru and Pip appear to be quite similar. Finally, the TiMOF nanoformulations are more likely candidates for therapeutic delivery than Zr-MOF nanoformulations because of better enhancing of both effects. For these reasons, we suggest that TiMOFs may be promising for developing DDSs for natural agents. Future investigations are needed to target specific neurodegenerative disease models.

## Conclusions

A novel anti-inflammatory and antioxidant nanoformulation consisting of MOFs loaded with Ru (a flavonoid) and/or Pip (an alkaloid) was developed. The MOF carrier particles were surface modified to yield Ti-MOFTS and Zr-MOFTS. Nanoformulations loaded with one of the agents as well as both together were compared with the natural agents without carrier. Paw edema and leukocyte migration activity were significantly more reduced in rats intraperitoneally injected with nanoformulations than with free Ru and/or Pip. The best results were obtained when rats were injected with a nanoformulation containing a mixture of single-drug nanoformulations of Ru and Pip. A similar trend was observed for the antioxidant effect. Overall, a high total loading content was achieved, at 17.11 ± 1.43% for Pip and 15.56 ± 1.24% for Ru loading into Ti-MOF. For dual loading, Ti-MOFs could incorporate about 14% of Ru and about 13% of Pip, demonstrating not only the potential to load two agents but also a high loading capacity at ∼27%. The silane-modified MOFs showed a sustained release effect within 48 hours compared to un-modified MOFs where a fast release within 24 hours was observed. Release of the drugs from the silane-modified MOF carriers followed two stages, suggesting mixed release kinetics at pH 7.4. The first stage followed zero-order kinetics for the first 12 hours, and the second stage was a stable release from 12 up to 48 hours, fitting the Korsmeyer–Peppas model. The prepared nanoformulations showed predictable kinetic release patterns and important enhancement in anti-inflammatory and antioxidant activities over free natural agents in the *in vivo* studies.

## Supplementary Material

Supplemental Material
