# Supplementary material for: Anti-inflammatory and antioxidant effects of nanoformulations composed of metal-organic frameworks delivering rutin and/or piperine natural agents
Source: Drug Deliv. 2021 Jul 13;28(1):1478–95. doi: 10.1080/10717544.2021.1949073 (PMC8280904; doi:10.1080/10717544.2021.1949073)
Supplement: Supplemental Material [file IDRD_A_1949073_SM8426.docx]

**Anti-inflammatory and antioxidant effects of nanoformulations composed of metal–organic frameworks delivering rutin and/or piperine natural agents**

**Khaled AbouAitah^1,2^*, Iman M. Higazy^3^, Anna Swiderska-Sroda^1^, Reda M. Abdelhameed^4^, Stanislaw Gierlotka^1^,** **Tarik A. Mohamed ^5^, UrszulaSzałaj^1,6^, and Witold Lojkowski^1^***

^1^Laboratory of Nanostructures and Nanomedicine, Institute of High Pressure Physics, Polish Academy of Sciences, Sokolowska 29/37, 01-142 Warsaw, Poland.

^2^Medicinal and Aromatic Plants Research Department, Pharmaceutical and Drug Industries Research Division, National Research Centre (NRC), 33 El-Behouth St, Dokki, Giza, 12622, Egypt.

^3^Department of Pharmaceutical Technology, Pharmaceutical and Drug Industries Research Division, National Research Centre (NRC), 33 El-Behouth St, Dokki, Giza, 12622, Egypt.

^4^Applied Organic Chemistry Department, Chemical Industries Research Division, National Research Centre (NRC), 33 El-Behouth St, Dokki, Giza, 12622, Egypt.

^5^Chemisrty of Medicinal plants Department, Pharmaceutical and Drug Industries Research Division, National Research Centre (NRC), 33 El-Behouth St, Dokki, Giza, 12622, Egypt.

^6^Faculty of Materials Engineering, Warsaw University of Technology, Wołoska 41, 02-507 Warsaw, Poland.

***** Correspondence:

K.A.: k.abouaitah@labnano.pl; the current address: National Research Centre, Dokki, Egypt; W. L.: w.lojkowski@labnano.pl; Tel.: +48-22-888-0429 or +48-22-632-4302; Fax: +48-22-632-4218

**NMR analysis of rutin**

**Rutin**. Yellow amorphous powder, m.p. 190°C; ^1^H-NMR (300 MHz, d-DMSO, δ_H_) 7.54 (1H, dd, J = 9.0, 2.1 Hz, H-6ʹ), 7.57 (1H, d, J = 2.1 Hz, H-2ʹ), 6.85 (1H, d, J = 9 Hz, H-5ʹ), 6.39 (1H, d, J = 2.1 Hz, H-8), 6.20 (1H, d, J = 2.1 Hz, H-6), 5.35 (1H, d, J = 7.5 Hz, H-1ʹʹ), 4.40 (1H, d, J = 2.0 Hz, H-1ʹʹʹ) 1.01 (3H,d, J= 6.3 Hz, CH_3_-rha) 3.16-3.65 (m, the rest sugar of glucose and rhamnose), ^13^C-NMR (100 MHz, CD_3_OD, δc) δ 180.0 (C-4), 166.1 (C-7), 162.5 (C-5), 159.1 (C-9), 158.7(C-2), 150.2 (C-4’), 146.1 (C-3ʹ), 135.1 (C-3), 123.1 (C-1ʹ), 122.5 (C-6ʹ), 118.0(C-5ʹ), 116.1(C-2ʹ), 105.5 (C-10), 102.4 (C-1ʹʹ), 101.0 (C-1ʹʹʹ), 100.1 (C-6), 95.0 (C-8), 78.2 (C-3ʹʹ), 77.2 (C-5ʹʹ), 75.7 (C-2ʹʹ), 71.4 (C-4ʹʹ), 73.9 (C-4ʹʹʹ), 72.2 (C-3ʹʹʹ), 72.1 (C-2ʹʹʹ), 69.7 (C-5ʹʹʹ), 68.6 (C-6ʹʹ), 18.0 (C-6ʹʹʹ).

**HPLC analysis of rutin**


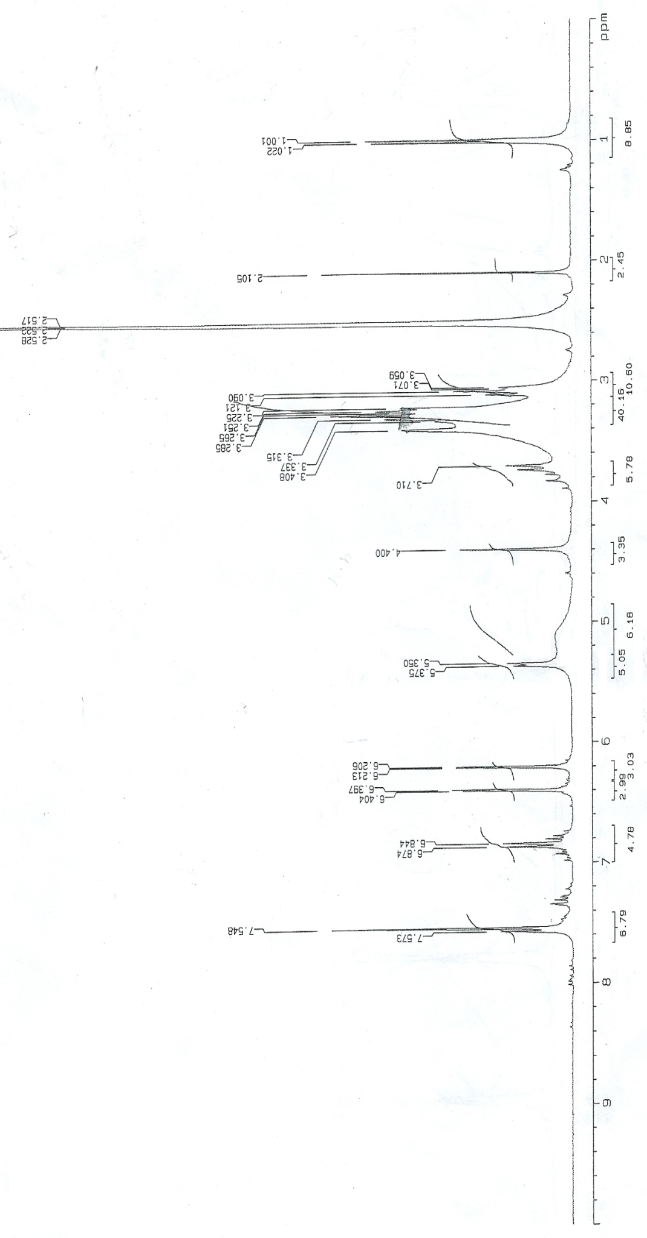
HPLC analysis was carried out using an Agilent 1260 series. The separation was carried out using ODS column (4.6 mm×250mm). The mobile phase used was methanol : H_2_O (70:30) at a flow rate of 0.7 ml/min. Injection volume was 10 μl. Detection was carried out by monitoring the absorbance signals at 254 nm.

**Figure S1.**^1^H NMR (DMSO-d6, 300 MHz) of Rutin

**
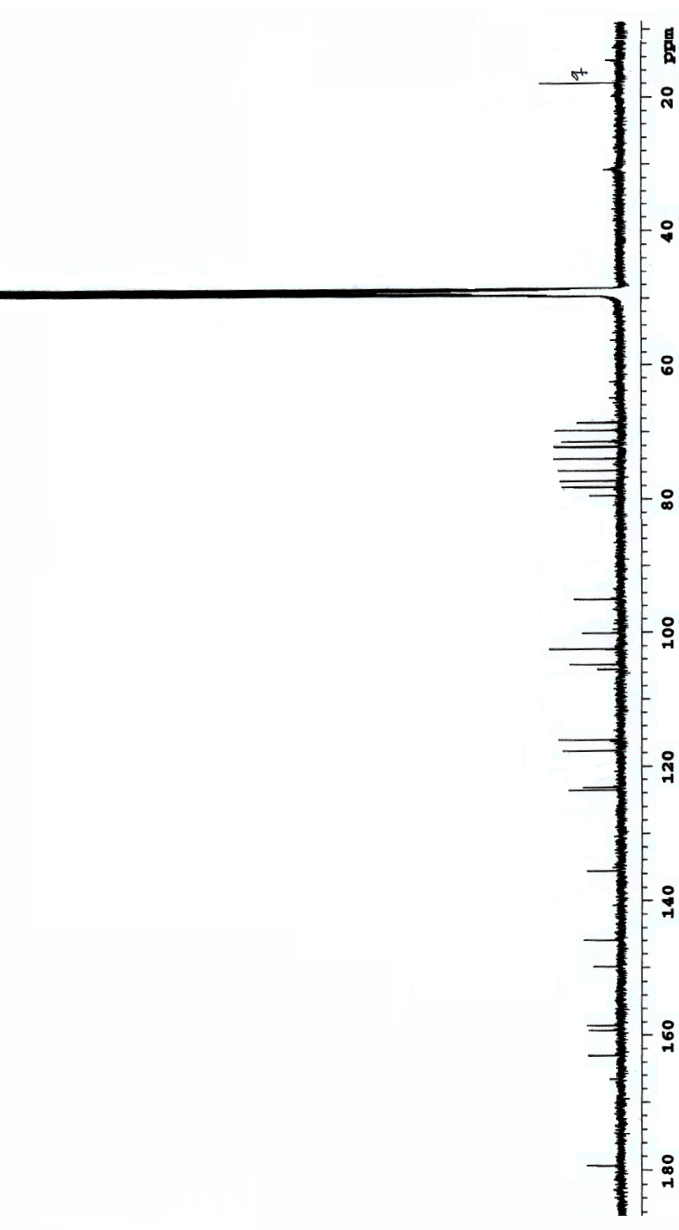
**

**Figure S2.**^13^C NMR (DMSO-d6, 125 MHz) of Rutin


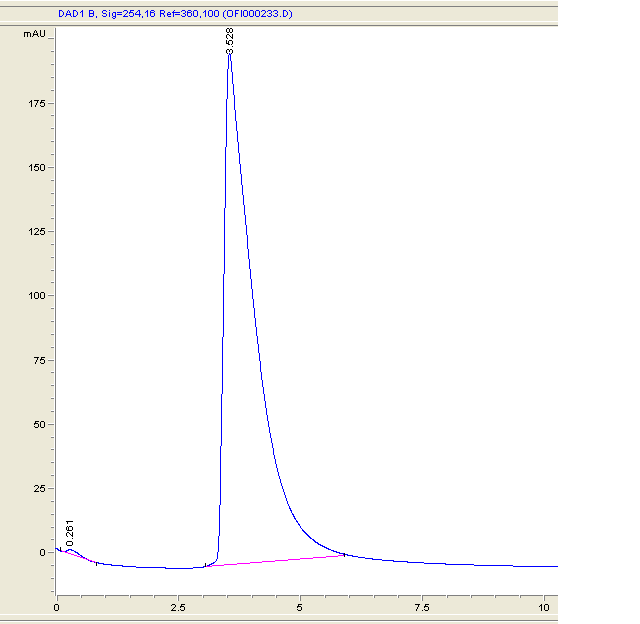


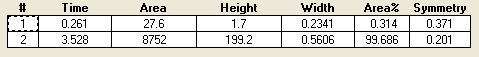


Figure S3. HPLC chromatogram of rutin HPLC, high-performance liquid chromatography.

**Table S1**. Elemental analysis of the materials studied.

| **Sample** | **Ti*** | **Zr*** | **Si*** | **C** | **H** | **N** |
| --- | --- | --- | --- | --- | --- | --- |
|  |  |  |  |  |  |  |
| ZrMOF  = Zr6O4(OH)4(C9H4O6)​6 |  | 28.39  ±3.61 | - | 33.13  (33.64) | 1.71  (1.46) | - |
| ZrMOFTS  = Zr6O4(OH)4(C9H4O6)​6(C16H19Si)0.1 | - | 27.97  ±1.61 | 0.14  ±.01 | 34.78  (34.37) | 1.61  (1.55) | - |
| TiMOF  _=_ Ti8O8(OH)_4_(C_8_H_5_O_4_N)_6_ | 23.16  ±1.43 | - | - | 34.77  (34.86) | 2.44  (2.07) | 5.17 (5.08) |
| TiMOFTS  _=_ Ti8O8(OH)4(C8H5O4N)4(C24H23SiO4N)2 | 17.97  ±2.57 | - | 2.64  ±0.25 | 45.78 (45.10) | 3.62 (3.31) | 4.05 (3.94) |

*The metal ratio calculated from Atomic absorption spectroscopy (AAS) and the results are expressed as mean ±SD for determination of 5 crystals. C, H, N percentages were measured for 3 samples and calculated value between brackets.

**Table S2.** *Invitro* release of rutin (Ru) and piperine (Pip) at pH 7.4 from various Zr and Ti-based MOF nano-carriers

| **Time (h)** | **Mean cumulative amount of drug released from various formulations (% ± SD)** | | | |
| --- | --- | --- | --- | --- |
|  | **ZrMOF-Ru** | **ZrMOF-Pip** | **TiMOF-Ru** | **TiMOF-Pip** |
| **0** | 0.00 | 0.00 | 0.00 | 0.00 |
| **1** | 3.57 ± 0.18 | 4.10 ± 0.36 | 5.28 ± 0.49 | 4.70 ± 0.33 |
| **2** | 8.23 ± 0.72 | 8.03 ± 0.75 | 9.13 ± 0.85 | 7.80 ± 0.58 |
| **3** | 14.90 ± 0.98 | 13.55 ± 1.00 | 16.40 ± 0.90 | 12.64 ± 1.05 |
| **4** | 22.17 ± 1.11 | 20.68 ± 1.13 | 25.39 ± 1.06 | 21.48 ± 1.34 |
| **5** | 33.19 ± 1.26 | 30.93 ± 2.01 | 38.21 ± 2.02 | 30.67 ± 1.36 |
| **6** | 46.11 ± 1.84 | 41.57 ± 3.15 | 50.16 ± 1.76 | 44.01 ± 1.72 |
| **8** | 64.42 ± 2.16 | 60.33 ± 1.84 | 70.82 ± 2.43 | 62.18 ± 2.25 |
| **12** | 80.03 ± 3.03 | 78.98 ± 1.55 | 88.60 ± 4.01 | 82.23 ± 1.69 |
| **24** | 95.70 ± 3.88 | 93.49 ± 3.46 | 98.51 ± 2.99 | 96.90 ± 3.59 |
| **Kinetic Model** | Korsmeyer-Peppas | | | |
| **R^2^** | 0.9990 | 0.9993 | 0.9991 | 0.9992 |
| **Release Efficiency (MRE)**  **(% ± SD)** | 64.96 ± 1.33 | 63.06 ± 0.70 | 70.08 ± 1.62 | 65.38 ± 0.87 |
| **Mean Release Rate (MRR) (%/h ± SD)** | 3.99 ± 0.07 | 3.90 ± 0.12 | 4.10 ± 0.23 | 3.95 ± 0.09 |
| **Mean Release Time (MRT) (h)** | 7.71 ± 0.40 | 7.81 ± 0.28 | 6.93 ± 0.55 | 7.71 ± 0.14 |
